# Chronic psychological stress disrupts liver homeostasis by dysregulating oxidative phosphorylation via the PI3K/AKT/FoxO3a axis

**DOI:** 10.1016/j.isci.2026.115389

**Published:** 2026-03-18

**Authors:** Yue Wang, Rongjie Zhao, Ziyin Yuan, Lina Zhai, Xin Ning, Man Lv, Zihui Jin, Haroon Iqbal, Uzair Ur-Rehman, Zhou Yi, Wangkai Chen, Baichuan Wang, Haoyue Qianjiang, Lihong Li, Huacheng Luo, Run Xiao

**Affiliations:** 1 Medical College of Tianjin University, Tianjin University, Tianjin 300072, China; 2 Zhejiang Cancer Hospital, Hangzhou Institute of Medicine, Chinese Academy of Sciences, Hangzhou, Zhejiang 310022, China; 3 Department of Gynecologic Oncology, Zhejiang Cancer Hospital, Hangzhou, Zhejiang 310022, China; 4 College of Pharmaceutical Science, Zhejiang University of Technology, Hangzhou 310014, China; 5 Harbin Medical University Cancer Hospital, Harbin 150081, China; 6 School of Life Science, Tianjin University, Tianjin 300072, China; 7 Eye Hospital, School of Ophthalmology and Optometry, Wenzhou Medical University, Wenzhou 325027, Zhejiang, China; 8 Department of Acupuncture, the Second Affiliated Hospital of Zhejiang Chinese Medical University, Hangzhou 310005, China; 9 Jinhua Academy of Zhejiang Chinese Medical University, Jinhua 310053, China

**Keywords:** hepatology, molecular biology

## Abstract

Epidemiological evidence indicates that chronic psychological stress correlates with the morbidity and mortality of chronic liver disease. However, the underlying mechanisms remain unclear. We established a chronic restraint stress (CRS) mouse model to simulate emotional stress. Histological and biochemical analyses showed marked hepatocyte vacuolization, increased transaminase levels, and apoptosis, signifying injury. Single-cell RNA sequencing showed that psychological stress suppresses oxidative phosphorylation (OXPHOS), consistent with mitochondrial abnormalities identified by electron microscopy. Forkhead box O (FoxO)3a was identified as a key transcription factor mediating CRS-induced OXPHOS inhibition, particularly in periportal hepatocytes (zone 1). Furthermore, FoxO3a-driven epigenetic silencing contributed to OXPHOS reduction. In upstream signaling, the phosphoinositide 3-kinase (PI3K)/protein kinase B (AKT) pathway was suppressed, leading to enhanced FoxO3a activity. Collectively, these findings reveal that chronic stress disrupts hepatocyte homeostasis via PI3K/AKT/FoxO3a-mediated OXPHOS dysregulation. This study deepens the understanding of psychological stress-induced liver dysfunction and highlights impaired mitochondrial oxidative metabolism as a potential therapeutic target for psychological intervention in liver disorders.

## Highlights

• Chronic stress disrupts hepatocyte homeostasis with mitochondrial dysfunction
• FoxO3a acts as a key regulator of stress-induced liver dysfunction
• Elevated FoxO3a impairs periportal hepatocyte OXPHOS via epigenetic gene silencing
• Chronic stress enhances hepatocyte FoxO3a activity via PI3K/AKT pathway suppression

## Graphical abstract

**Figure:**
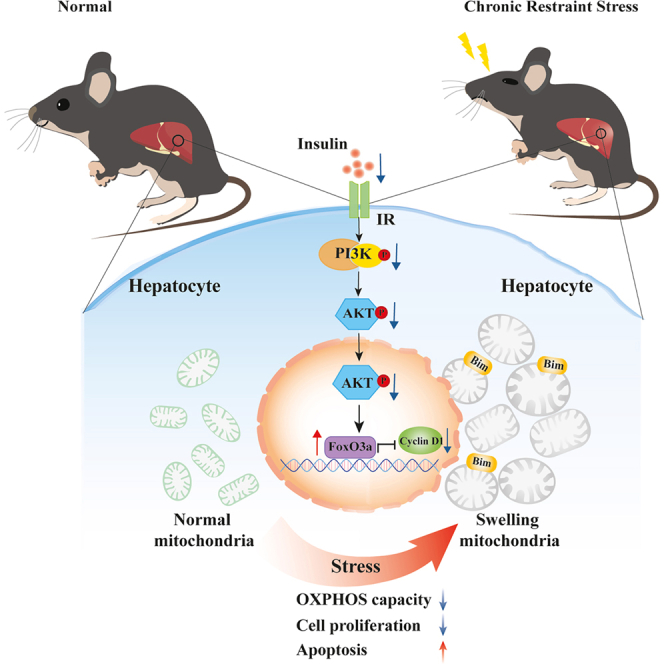

## Introduction

Chronic psychological stress not only has a profound impact on psychological well-being but also causes damage to physical health, potentially leading to the dysregulation of physiological processes and the development of diseases. Exposure to negative external stimuli generates emotional stress, which has been linked to impaired liver function and contributes to the pathogenesis of liver-related diseases.^1^ Population-based studies have demonstrated a significant correlation between psychological factors and liver dysfunction.^2^^,^^3^ Epidemiological studies have revealed a strong association between negative emotions, such as depression and anxiety, and the development of liver diseases. A study involving 405,073 participants from the UK demonstrated that loneliness and social isolation were associated with an increased incidence of non-alcoholic fatty liver disease (NAFLD), independent of other risk factors.^4^ In addition, another prospective study from the UK showed that the level of psychological stress was positively correlated with the severity of chronic liver disease.^5^ These psychological disturbances may contribute to the progression of conditions such as hepatocellular carcinoma (HCC) and cirrhosis, potentially mediated by pathways associated with chronic stress.^6^^,^^7^ However, the underlying mechanisms by which psychological stress affects liver physiology and disease remain incompletely understood.

The liver is a vital metabolic organ with diverse functions, and its spatial organization into distinct zones enables an efficient division of labor to maintain homeostasis.^8^ Within the hepatic lobule, hepatocytes are typically arranged into three distinct zones: zone 1, adjacent to the portal vein; zone 3, proximate to the central vein; and zone 2, positioned in the intervening region. Hepatocytes in zone 1—advantaged by the oxygen- and nutrient-rich blood supply from the portal vein and hepatic artery—are primarily responsible for gluconeogenesis and beta-oxidation. Conversely, hepatocytes in zone 3, which exist in a microenvironment characterized by lower oxygen tension and nutrient availability near the central vein, are more active in glycolysis and lipogenesis.^8^ The structural and metabolic foundations allow for differential susceptibility to damage, driven by oxygen tension, metabolic activity, and toxin exposure.

The forkhead box O (FoxO) protein family, consisting of FoxO1, FoxO3a, FoxO4, and FoxO6 in mammals, acts as critical transcriptional regulators that orchestrate cellular and organismal adaptive responses to diverse stressors and environmental changes.^9^^,^^10^ These proteins regulate diverse cellular processes, including lifespan, cell survival, and cellular homeostasis.^11^^,^^12^^,^^13^ FoxO3a, in particular, plays a pivotal role in regulating metabolism and cell-cycle arrest by directing the transcription of specific downstream genes.^14^^,^^15^ Zhou et al. reported that behavioral stress activates FOXO3a in the cerebral cortex, linking it to behavioral disturbances.^16^ The role of FoxO3a in liver homeostasis in response to chronic psychological stress has not been defined.

Previous investigations have established that exposure to various xenobiotics and heavy metals, such as arsenic and copper, triggers hepatotoxicity through the dysregulation of immune responses and metabolic homeostasis.^17^^,^^18^ However, the precise molecular mechanisms by which chronic psychological stress impairs liver function remain elusive. Exploring the underlying mechanisms of liver function homeostasis under psychological stress provides valuable insights into the complex interplay between psychological stress and liver physiology. In this study, we investigated the characteristics of liver damage induced by psychological stress using a chronic restraint stress (CRS) model. Our findings revealed mitochondrial dysfunction in hepatocytes as a key feature of CRS-induced liver damage and elucidated the role of the PI3K/AKT/FoxO3a pathway in disrupting liver physiology and homeostasis.

## Results

### CRS leads to depressive-like behaviors in mice

**Figure 1:**
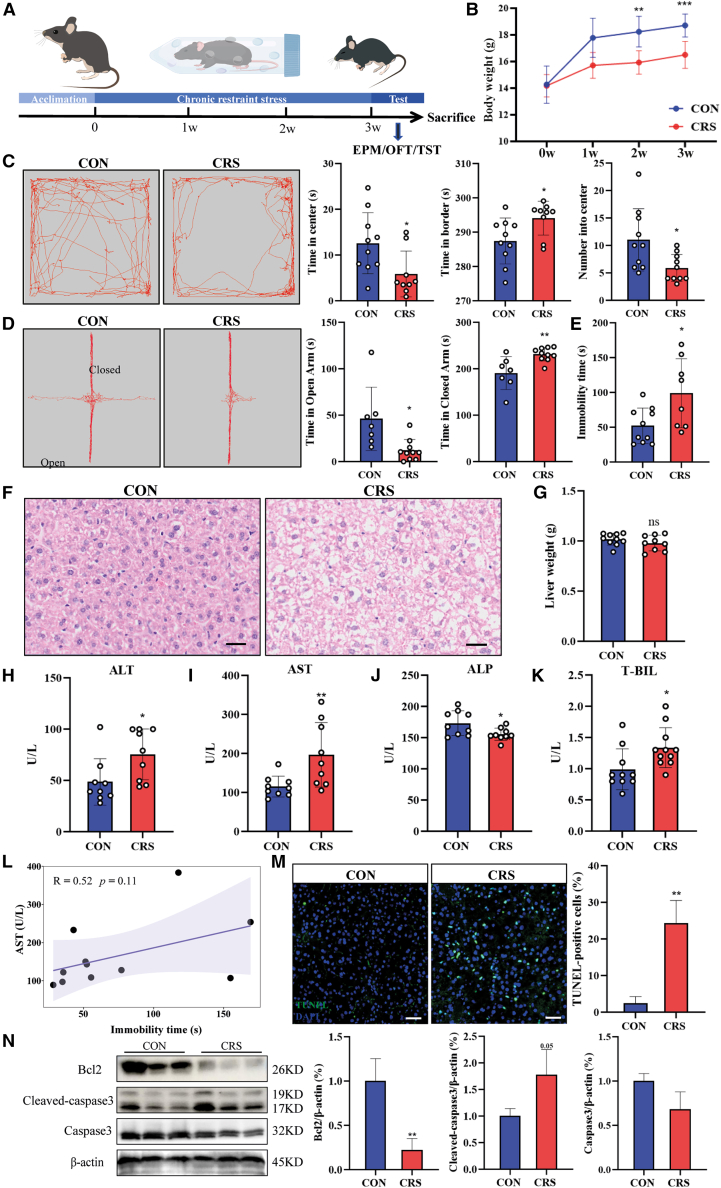
CRS triggers hepatic dysfunction in mice (A) Scheme of the experimental design. EPM, elevated plus maze; OFT, open field test; TST, tail suspension test. (B) Body weight changes in mice during CRS model establishment. (C) Representative tracks and statistics for the OFT. (D) Representative tracks and statistics for the EPM. (E) Immobility time of CON and CRS mice in the TST (*n* = 10/group). (F) H&E staining of liver sections from CON and CRS mice. Scale bars, 50 μm. (G) Comparison of liver tissue weight between CON and CRS mice. (H–K) Measurement of serum levels of hepatic function markers: (H) alanine aminotransferase (ALT), (I) aspartate aminotransferase (AST), (J) alkaline phosphatase (ALP), and (K) total bilirubin (T-BIL) (*n* = 10). (L) Correlation between immobility time and AST level. (M) TUNEL staining of liver slices. Quantification of TUNEL staining (*n* = 3 mice/group). (N) Western blot analyses of apoptosis-related proteins in the livers of CON and CRS mice. Data are represented as means ± SD. *p* values were calculated using a two-tailed unpaired Student’s *t* test. ∗*p* < 0.05, ∗∗*p* < 0.01, ∗∗∗*p* < 0.001.

To establish a psychological stress model potentially associated with liver dysfunction, we employed the widely recognized CRS method. Female C57/BL6 mice were restrained for 6 h daily over 21 consecutive days. Body weight was measured weekly, while anxiety- and depressive-like behaviors were assessed using the elevated plus maze (EPM), open field test (OFT), and tail suspension test (TST) at the end of the 3-week stress treatment period (Figure 1A). Compared to the control (CON) group, CRS mice displayed decreased weight gain (Figure 1B), but there was no significant change in the liver-to-body weight ratio (Figure S1A). Meanwhile, heart-, lung-, and kidney-to-body weight ratios showed no significant changes, although the spleen-to-body weight ratio was decreased in CRS mice (Figures S1B–S1E).

Accumulating evidence indicates that stress is closely associated with mood disorders. To investigate this relationship, mice subjected to CRS treatment underwent a series of behavioral tests evaluating anxiety and depression. In the OFT (Figure 1C), CRS mice exhibited reduced interest in exploring the central area compared to CON mice and showed a preference for activity in the border region. Consistently, CRS mice spent less time in the open arms during the EPM test (Figure 1D). Overall, these tests showed that chronically stressed mice exhibited a significant preference for safe regions. To further confirm the impact of chronic stress, we performed the TST, which revealed that CRS mice had significantly increased immobility time (Figure 1E), indicating that mice subjected to chronic stress gave up struggling more quickly. Collectively, mice undergoing 21 days of restraint stress developed anxiety- and depressive-like behaviors. The phenotype of the CRS model closely resembles psychological responses in humans, making it suitable for exploring stress-induced effects on liver function.

### CRS results in hepatic dysfunction and apoptosis

To explore the CRS on the liver, hematoxylin and eosin (H&E) staining was performed on the liver tissues from CON and CRS mice. Notably, a large area of cytoplasmic vacuolation was observed in hepatocytes of CRS mice (Figure 1F), while Oil Red O (ORO) staining was performed to rule out the accumulation of lipids (Figure S1F). Periodic acid-Schiff (PAS) staining results showed that glycogen deposition was dramatically reduced in the CRS group, implying insufficient energy supply and metabolic disorder (Figure S1G). Thus, this is likely diffuse hydropic degeneration in hepatocytes caused by chronic stress.^19^ In the CRS group, liver weight showed a trend of reduction in comparison to the CON group, but there was no significant difference between the two groups (Figure 1G). Liver function was assessed using serum levels of alanine aminotransferase (ALT), aspartate aminotransferase (AST), alkaline phosphatase (ALP), and total bilirubin (T-BIL). Compared to the CON group, serum levels of ALT, AST, and T-BIL were significantly elevated in CRS mice, while the serum level of ALP decreased (Figures 1H–1K), indicating liver dysfunction in CRS mice. To test whether there is sex difference in CRS-induced hepatic dysfunction, we established a CRS model in male C57BL/6 mice. Consistent with the observations in female mice, male CRS mice showed a significant reduction in body weight gain (Figure S1H). Histopathological examination of liver tissue via H&E staining similarly revealed marked vacuolization (Figure S1I). Behaviorally, the mice exhibited a pronounced increase in immobility time in the TST (Figure S1J), reflecting a depressive-like phenotype. This structural damage was corroborated by a significant impairment in hepatic functional integrity, as indicated by elevated serum levels of the transaminases ALT and AST, and abnormal levels of ALP and T-BIL (Figure S1K). These data demonstrate that the hallmark features of CRS-induced liver injury were effectively reproduced in male mice, suggesting that the pathophysiological consequences of the model are not sex-dependent. Further analysis via Pearson correlation demonstrated a moderate positive correlation between TST immobility time and serum AST concentrations (R = 0.52, *p* = 0.11), indicative of a distinct positive trend that approached statistical significance. This finding thereby supports a potential link between individual variability in stress responses and the extent of liver dysfunction (Figure 1L). Increasing evidence suggests that various types of stress can induce apoptosis-related liver dysfunction.^20^^,^^21^^,^^22^ To assess hepatic apoptosis, we performed TUNEL staining on liver sections, which showed that CRS dramatically increased the number of TUNEL-positive cells compared to the CON group (Figure 1M). CRS-induced apoptosis in the liver was further confirmed by western blot, which revealed increased expression of cleaved caspase-3 and decreased level of Bcl-2 (Figure 1N). Notably, the proapoptotic protein Bim was elevated in hepatocytes of CRS mice (Figure S1L). Collectively, these data suggest that CRS leads to liver injury and hepatic apoptosis.

### CRS leads to metabolic disorder

**Figure 2:**
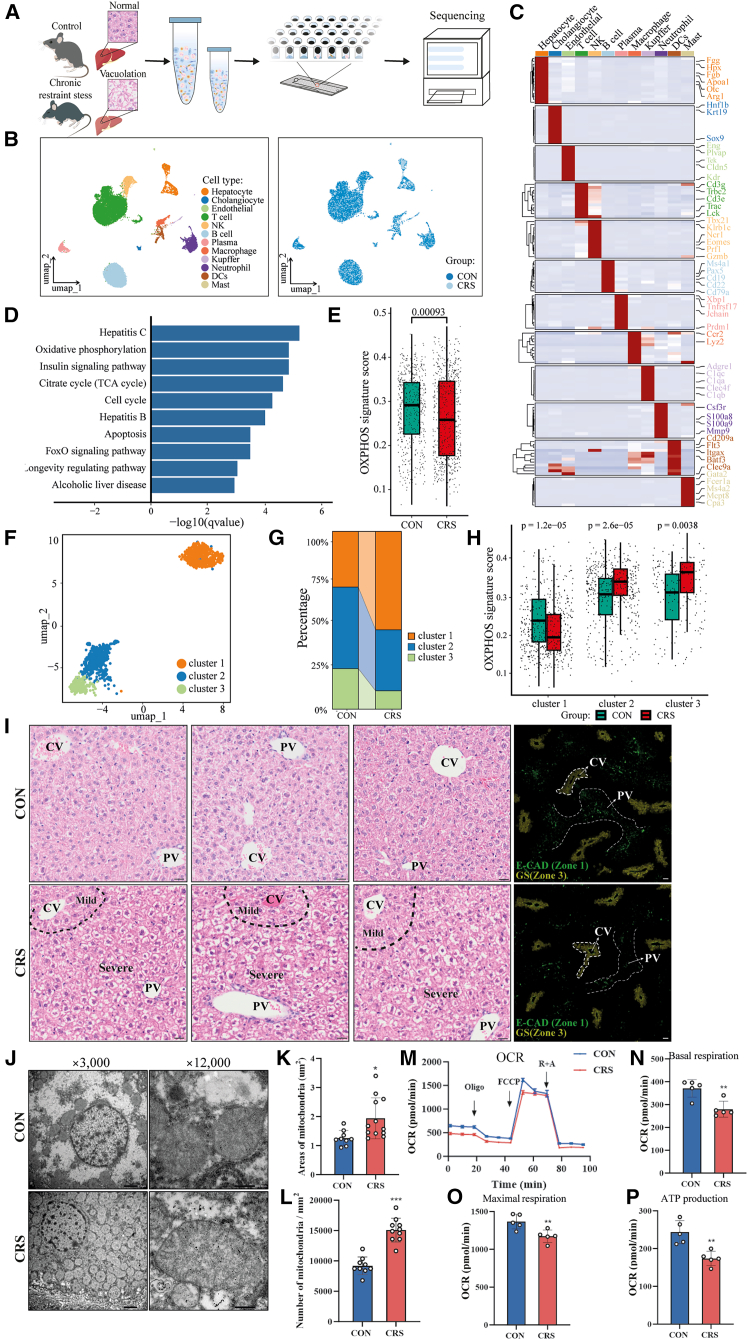
CRS induces dysregulation of mitochondrial oxidative phosphorylation in hepatocytes (A) Schematic of single-cell RNA sequencing (scRNA-seq) of CRS mouse livers. (B) UMAP visualization of hepatic tissues components, color-coded by tissue origin (healthy vs. CRS hepatic tissues). (C) Cell type-specific marker gene expression profiles displayed as a *Z* score-normalized heatmap. Annotated marker genes used for cell type annotation are indicated. (D) Dysregulated pathways enriched by differentially expressed genes (DEGs) between healthy and CRS-induced hepatocytes (q-value < 0.05). (E) Comparative analysis of oxidative phosphorylation (OXPHOS) pathway activity assessed by AUCell in hepatocyte populations. Boxplot elements: center line = median, box limits = first and third quartiles, whiskers = 1.5 × interquartile range (IQR). Statistical significance was determined by a two-sided Wilcoxon test. (F and G) Hepatocyte subcluster analysis and proportional distribution. Bar plot height represents relative subcluster abundance within each group. (H) OXPHOS pathway signature scores across hepatocyte subclusters stratified by tissue origin. Data were analyzed using two-sided Wilcoxon tests. (I) Representative images of liver histopathology by H&E staining and immunofluorescence. Immunofluorescence staining identifies the periportal (zone 1) and pericentral (zone 3) regions using markers for E-cadherin (E-CAD) and glutamine synthetase (GS), respectively. Scale bars, 50 μm. (J) Mitochondria morphology of CON and CRS hepatocytes was analyzed by TEM. Scale bars, 500 nm or 1 μm. (K and L) The number and size of mitochondria were summarized. (M) OCR analysis of hepatocytes was measured at multiple time points with consecutive administration of 1 μM oligomycin, 1 μM carbonyl cyanide-4-(trifluoromethoxy)phenylhydrazone (FCCP), and 1 μM rotenone and antimycin A. (N–P) The statistical results were presented for OCR assays (*n* = 3 mice/group). Data are represented as means ± SD. *p* values were calculated using a two-tailed unpaired Student’s *t* test. ∗*p* < 0.05, ∗∗*p* < 0.01, ∗∗∗*p* < 0.001.

To investigate the mechanism underlying CRS-mediated liver injury, we collected freshly isolated liver tissues from healthy CON and CRS mice and then performed single-cell RNA sequencing (scRNA-seq) analysis (Figure 2A). Uniform manifold approximation and projection (UMAP) visualization of 23,356 cells identified distinct cell clusters, which were annotated to known cell lineages using canonical marker genes.^23^^,^^24^^,^^25^ These included hepatocytes, cholangiocytes, immune cells (T cells, natural killer [NK] cells, B cells, plasma cells, macrophages, Kupffer cells, neutrophils, dendritic cells, and mast cells), and stromal cells (endothelial cells) (Figures 2B, 2C, S2A, and S2B). Given that CRS induced severe cytoplasmic vacuolation in hepatocytes (Figure 1F), we focused on identifying differentially expressed genes (DEGs) between CRS and CON hepatocytes, followed by Kyoto Encyclopedia of Genes and Genomes (KEGG) enrichment analysis. Enriched pathways included cell cycle, apoptosis, and FoxO signaling, indicating that CRS impacted hepatocyte transcription and growth (Figure 2D). Additionally, pathways associated with hepatitis B, hepatitis C, and alcoholic liver disease were enriched, suggesting that CRS-induced liver dysfunction may share molecular mechanisms with these diseases and confirming the negative regulatory effect of CRS on liver homeostasis (Figure 2D). Oxidative phosphorylation (OXPHOS) and the tricarboxylic acid (TCA) cycle—key to mitochondrial energy metabolism—were also enriched, highlighting that mitochondrial dysregulation may play an important role in CRS-mediated liver injury. Since the OXPHOS pathway was the most highly enriched, we compared OXPHOS signature scores between CRS- and CON mouse-derived hepatocytes and found that overall OXPHOS levels were markedly reduced in the CRS group (Figure 2E).

To delineate alterations in hepatocyte states underlying CRS-induced liver dysfunction, we performed a subclustering analysis, which revealed three distinct subpopulations (Figures 2F–2H and S3). Cluster 1 exhibited enriched expression of genes involved in amino acid/organic acid metabolism, fatty acid β-oxidation, bile acid secretion, urea cycle, and gluconeogenesis, consistent with the established periportal hepatocyte (zone 1), which are located adjacent to the portal vein.^26^ In contrast, clusters 2 and 3 shared biological processes related to lipid biosynthesis, cholesterol homeostasis, xenobiotic metabolism, and cellular oxidant detoxification. Cluster 2 uniquely expressed genes implicated in ω-fatty acid oxidation, peroxisome proliferator–activated receptor (PPAR) signaling, and cytochrome P450-mediated oxidation. The transcriptomic profiles of clusters 2 and 3 aligned with previously defined mid-lobular (zone 2) and pericentral (zone 3) hepatocytes.^26^^,^^27^

A comparative analysis of OXPHOS signature scores revealed significant suppression (*p* < 0.001) in cluster 1 hepatocytes from CRS mice compared to CON (Figure 2E). The proportion of these mitochondria-compromised cells expanded markedly in the CRS group (55% vs. 31% in CON), indicating sustained injury with potential compensatory adaptation (Figure 2G). Notably, CRS elicited opposing mitochondrial responses: it suppressed OXPHOS in cluster 1 while elevating it in clusters 2 and 3 (Figure 2H). Given the well-established metabolic zonation orchestrated by oxygen, nutrient, and hormone gradients,^8^ along with these subpopulation-specific pathway activations, we postulate that CRS induces zonally stratified liver injury, with periportal hepatocytes of zone 1 exhibiting greater vulnerability to stress stimuli than mid-lobular and pericentral populations. In support of this hypothesis, we conducted detailed histopathological analyses on liver sections from both CON and CRS groups. H&E staining and immunofluorescence (IF) revealed a clear spatial pattern of hepatocyte injury: hepatocyte vacuolization in the periportal region of zone 1 was significantly more severe than that in the pericentral region of zone 3 (Figure 2I).

The process of OXPHOS occurs in the mitochondria and generates ATP, the primary energy source for cellular processes. To study the role of mitochondria in CRS-induced hepatocyte dysfunction, transmission electron microscopy (TEM) was used for ultrastructure analysis, revealing mitochondrial abnormalities, including increased mitochondrial number, swelling, and instances of rupture (Figures 2J–2L). In addition, we measured the oxygen consumption rate (OCR) in mouse primary hepatocytes from CON and CRS mice (Figure 2M). Hepatocytes from the CRS group exhibited severely impaired OXPHOS, with significant reductions in basal respiration, maximal respiration, and ATP production (Figures 2N–2P). These data imply that CRS results in severe mitochondrial damage and disrupts mitochondrial function.

Structural mitochondrial defects frequently correlate with dysregulation of critical metabolic functions, including glucose homeostasis and lipid metabolism. Therefore, we performed glucose tolerance test (GTT) and insulin tolerance test (ITT) assays in both groups. Interestingly, CRS mice exhibited increased capability for glucose clearance and improved insulin sensitivity (Figures S4A and S4B). Additionally, we found that basal serum glucose levels were profoundly decreased (Figure S4C), consistent with the PAS staining results. Additionally, we measured serum levels of triglycerides (TG), high-density lipoprotein (HDL), low-density lipoprotein (LDL), and total cholesterol (T-CH) (Figures S4D–S4G). Among these, only LDL showed a slight decrease in the CRS group compared to the CON group. These findings align with the observations of mitochondrial damage and further confirm that CRS caused hydropic degeneration of hepatocytes.

### Hepatocyte FoxO3a expression is negatively correlated with OXPHOS capacity

**Figure 3:**
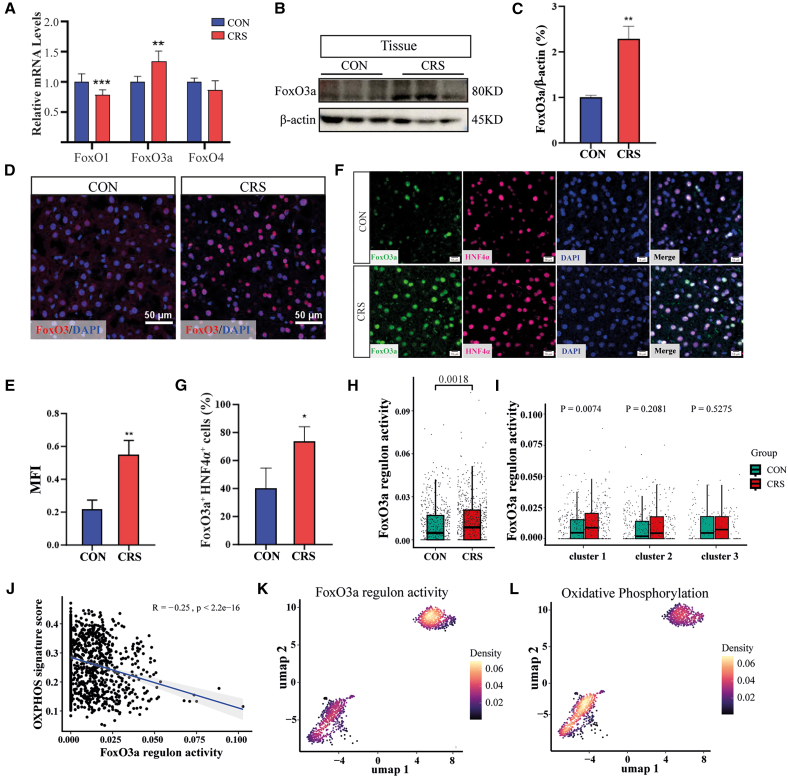
CRS-induced FoxO3a activation suppresses hepatocyte mitochondrial OXPHOS (A) FoxO1, FoxO3a, and FoxO4 mRNA levels in hepatocytes from CON and CRS mice (*n* = 5 mice/group). (B) Western blot analyses of FoxO3a in livers from CON and CRS mice. (C) Densitometric quantification of the FoxO3a/β-actin ratio from three independent western blot assays. (D) Immunofluorescent staining of FoxO3a in liver sections. Scale bars, 50 μm. (E) Mean fluorescence intensity (MFI) of FoxO3a^+^ cells was quantified. (F) Immunofluorescence staining of FoxO3a and HNF4α. (G) Percentage of FoxO3a^+^ HNF4α^+^ cells among total 4',6-diamidino-2-phenylindole (DAPI) positive cells was quantified. (H) Comparative analysis of FoxO3a regulon activity in hepatocytes from CON versus CRS donors, quantified using AUCell scoring. Boxplot elements: center line = median; box limits = 25th and 75th percentiles; whiskers = 1.5 × IQR. Significance was determined by a two-sided Wilcoxon test. (I) FoxO3a regulon activity distribution across hepatocyte subclusters from CON and CRS donors (two-sided Wilcoxon tests). (J) Pearson correlation analysis between FoxO3a regulon activity and OXPHOS pathway signature scores. (K and L) UMAP visualization of (K) FoxO3a regulon activity and (L) OXPHOS pathway signature scores in hepatocyte subclusters. A purple-to-yellow continuum indicates increasing scores. Data are represented as means ± SD. *p* values were calculated using a two-tailed unpaired Student’s *t* test. ∗*p* < 0.05, ∗∗*p* < 0.01, ∗∗∗*p* < 0.001.

As previously mentioned, the FoxO signaling pathway was significantly enriched in the DEGs of hepatocytes from the CRS group. Numerous studies have demonstrated that FoxO transcription factors serve as key mediators of adaptive responses to stress conditions, such as redox stress and nutrient stress.^28^ Therefore, we aimed to confirm whether FoxO signaling is involved in CRS-induced liver dysfunction and the inhibition of OXPHOS. To this end, we initially examined the mRNA expressional levels of FoxO transcription factors in hepatocytes. The expression of FoxO3a was significantly upregulated in the CRS group (Figure 3A). FoxO3a, a key multifunctional transcription factor, is known to critically regulate cellular processes such as cell cycle and stress resistance.^14^^,^^29^^,^^30^ In the CRS group, FoxO3a protein levels were significantly increased in liver tissues (Figures 3B and 3C). To confirm its expression in hepatocytes, IF staining was performed. HNF4a (also known as NR2A1), a master transcription factor in hepatocytes, serves as a marker for hepatocyte identity.^31^ We observed a significant increase in the proportion of FoxO3a^+^HNF4a^+^ cells, reinforcing the evidence that FoxO3a expression was upregulated (Figures 3D–3G).

We then constructed the FoxO3a regulon from our scRNA-seq data using pySCENIC and quantified its activity in hepatocytes by calculating the score with the AUCell algorithm. Consistent with the above results, overall FoxO3a regulon activity was profoundly elevated in the CRS group (Figure 3H). In the subgroup analysis, FoxO3a regulon activity in CRS hepatocytes of cluster 1 was significantly higher than that of the CON group (Figure 3I). Although clusters 2 and 3 showed no significant difference, there was a trend of increased FoxO3a regulon activity in CRS, supporting the notion that CRS leads to spatially stratified liver dysfunction. Consistent with the observed mitochondrial structural defects, FoxO3a regulon activity demonstrated a significant negative correlation with the OXPHOS enrichment score (Figure 3J). The negative correlation between OXPHOS and FoxO3a was also well supported at the subcluster level (Figures 3K and 3L).

### Knockdown of FoxO3a in CRS mice alleviated liver dysfunction

**Figure 4:**
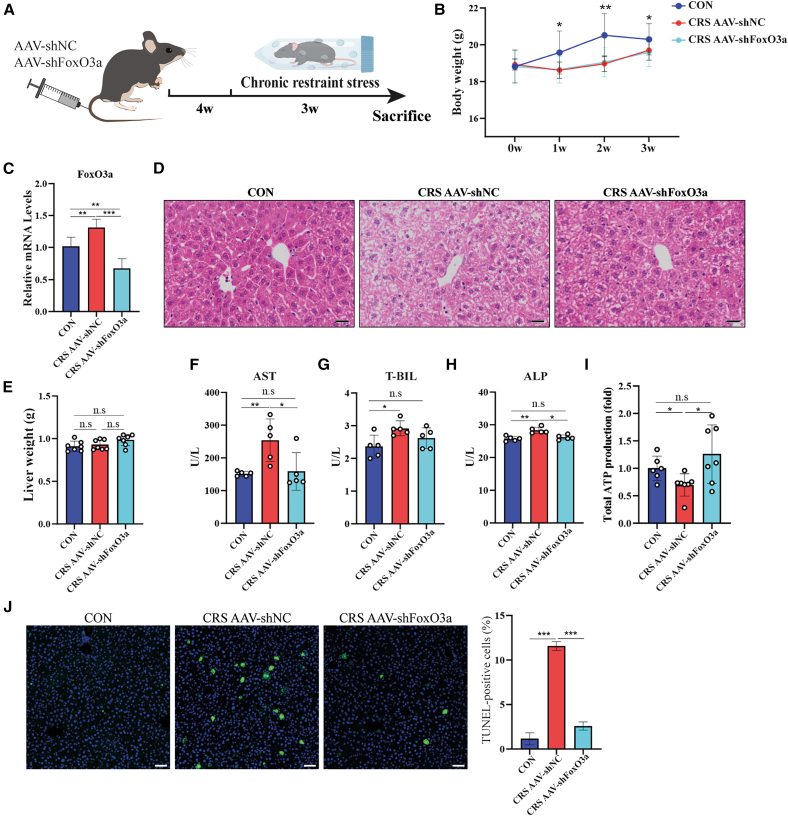
FoxO3a knockdown alleviates CRS-induced liver dysfunction (A) Schematic of the experimental design. Mice were administered with AAV encoding FoxO3a-specific shRNA (AAV-shFoxO3a) or a scramble sequence, followed by a 3-week CRS protocol. (B) Changes in body weight gain among different experimental groups. (C) Relative mRNA levels of FoxO3a among the three groups. (D) Representative images of H&E staining of liver tissue. Scale bars, 50 μm. (E) Liver weight changes among the three groups (*n* = 7). (F–H) Assessment of liver function indicators by measuring serum levels of (F) AST (aspartate aminotransferase), (G) T-BIL (total bilirubin), and (H) ALP (alkaline phosphatase). (I) Measurement of ATP levels among the three groups. (J) TUNEL staining of liver secions. Quantification of TUNEL staining (*n* = 3 mice/group). Scale bars, 50 μm. Statistical significance was determined by a two-tailed unpaired Student’s *t* test (∗*p* < 0.05, ∗∗*p* < 0.01, ∗∗∗*p* < 0.001).

To strengthen the evidence for the role of FoxO3a in CRS-induced liver dysfunction, we performed a liver-specific knockdown of FoxO3a by administering an adeno-associated virus-delivered short hairpin RNA (AAV-shFoxO3a), followed by a 3-week CRS protocol (Figure 4A). Both the CRS AAV-shNC and CRS AAV-shFoxO3a groups exhibited decreased weight gain compared to the CON group (Figure 4B). This approach achieved liver-specific knockdown of FoxO3a. Successful hepatic-specific knockdown was confirmed via qPCR (Figure 4C). As expected, FoxO3a deficiency mitigated cytoplasmic vacuolation induced by CRS (Figure 4D), without significantly affecting total liver weight among the three groups (Figure 4E). Furthermore, the CRS-induced elevations in hepatic dysfunction markers were largely reversed upon FoxO3a knockdown (Figures 4F–4H). Accompanied by the improved hepatic structure and function, ATP production in liver tissue was successfully recovered after FoxO3a knockdown (Figure 4I). Furthermore, TUNEL staining revealed that FoxO3a silencing markedly reduced CRS-induced hepatocyte apoptosis (Figure 4J). Collectively, these data further confirm that FoxO3a plays a vital role in mediating CRS-induced abnormalities in hepatic metabolism and function.

### CRS impairs hepatic OXPHOS through FoxO3a-driven epigenetic silencing

**Figure 5:**
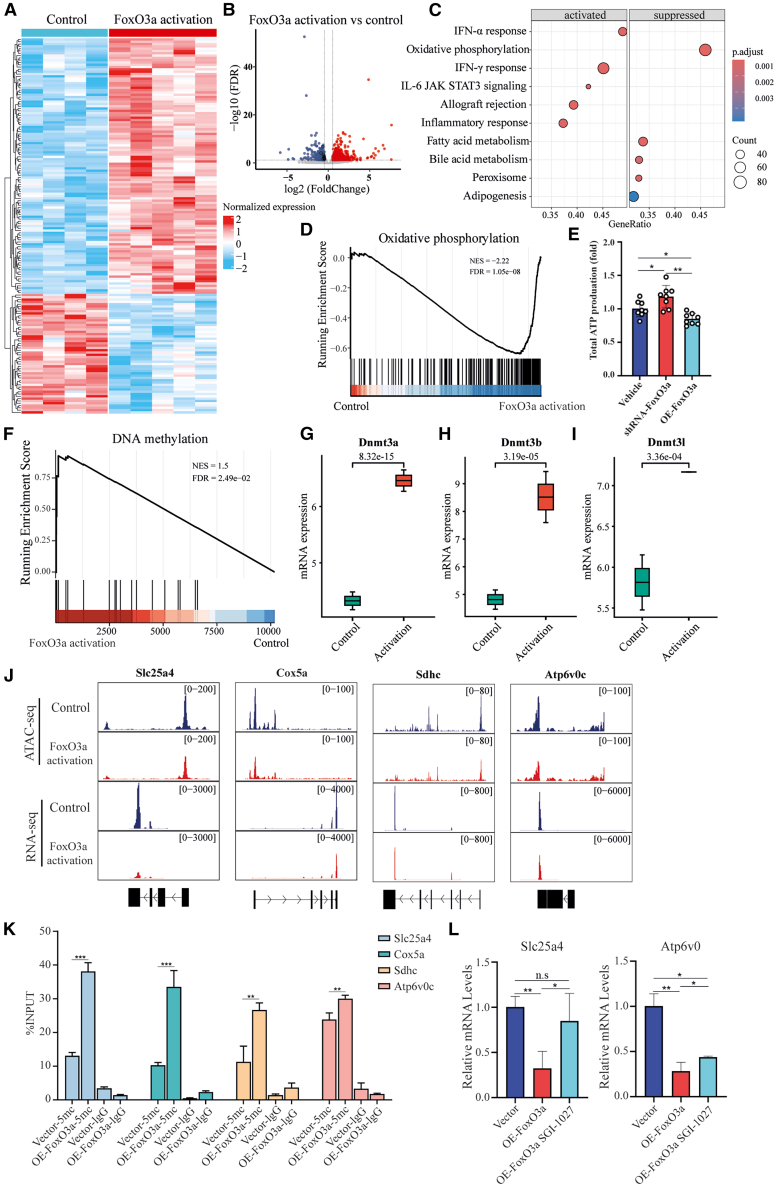
CRS impairs hepatic OXPHOS through FoxO3a-driven epigenetic silencing (A) Heatmap of DEG expression profiles in FoxO3a-activated versus wild-type hepatic tissues. A blue-to-red continuum depicts increased expression abundance. (B) Volcano plot of differentially expressed genes (DEGs) in FoxO3a-activated versus wild-type murine hepatic tissues (36-week cohort). Thresholds: false discovery rate (FDR) < 0.05, |log2 FC| > 0.5. Red and blue indicate upregulation and downregulation, respectively, in FoxO3a-activated tissue. (C) Top 10 significantly altered pathways (q-value < 0.05) in FoxO3a-activated hepatic tissues. Bubble size represents the number of enriched genes. Redness indicates significance. (D) Gene set enrichment analysis (GSEA) plot of DEGs in the Hallmark OXPHOS pathway. NES, normalized enrichment score. (E) Measurement of ATP levels in AML-12 cells with knockdown or overexpression of FoxO3a. (F) GSEA plot of DEGs in the Reactome DNA methylation pathway. (G–I) Comparative analysis of (G) Dnmt3a, (H) Dnmt3b, and (I) Dnmt3l expression between FoxO3a activation and control groups. Boxplot elements: center line = median, box limits = first and third quartiles, whiskers = 1.5 × interquartile range (IQR). *p* values were calculated using a two-tailed Wald test generated by DESeq2. (J) Genome browser views of the indicated sequencing results at representative loci upon FoxO3a activation. *n* = 2 independent biological replicates. (K) Methylated DNA immunoprecipitation quantitative PCR (MeDIP-qPCR) analysis showing 5-methylcytosine (5-mC) levels at the promoters of *Slc25a4*, *Cox5a*, *Sdhc*, and *Atp6v0c* in control vs. FoxO3a-overexpressing AML-12 cells. (L) mRNA levels of *Atp6v0c* and *Slc25a4* in AML-12 cells transfected with FoxO3a-overexpressing vectors followed by treatment with SGI-1027. Data are presented as means ± SD from three independent experiments. Statistical significance was determined by a two-tailed unpaired Student’s *t* test (∗*p* < 0.05, ∗∗*p* < 0.01, ∗∗∗*p* < 0.001).

To further confirm the regulatory relationship between FoxO3a and OXPHOS, we analyzed and compared RNA-seq data from mouse livers with high hepatic FoxO3a expression.^32^^,^^33^ Compared to the CON group, FoxO3a activation significantly altered the hepatic transcription profile, with 355 upregulated genes and 252 downregulated genes (Figures 5A and 5B). Hallmark pathway enrichment analysis revealed that FoxO3a activation significantly inhibits pathways related to OXPHOS, fatty acid metabolism, bile acid metabolism, and lipid synthesis (*q* < 0.05) (Figure 5C). Notably, OXPHOS ranked as the top inhibited pathway in 36-week-old mice (*q* < 0.0001) (Figures 5D and S6A), while it ranked fourth in 6-week-old mice (*q* = 0.0709) (Figures S5A–S5D, S6A, and S6B). This suggests that FoxO3a-mediated OXPHOS suppression is a progressively worsening process, highlighting the key role of FoxO3a in mitochondrial dysfunction and CRS-mediated hepatic injury. To further validate the role of FoxO3a in regulating OXPHOS, we overexpressed or knocked down the expression of FoxO3a in AML-12, which are widely used as mouse liver hepatocytes. Consistently, overexpression of FoxO3a led to a significant reduction in ATP levels, while its knockdown resulted in an increase in ATP levels. These findings suggest that FoxO3a plays a significant role in suppressing OXPHOS in CRS mice (Figure 5E).

To elucidate the mechanisms by which FoxO3a suppresses OXPHOS, we performed Reactome pathway enrichment analysis and identified significant enrichment of the DNA methylation pathway (Figure 5F). Most genes involved in this pathway, including Dnmt3a, Dnmt3b, and Dnmt3l, were upregulated following FoxO3a activation (Figures 5G–5I), suggesting the involvement of epigenetic control in transcriptional regulation. Furthermore, we observed a significant reduction in chromatin accessibility at key OXPHOS-related genes (*Slc25a4*, *Cox5a*, *Sdhc*, and *Atp6v0c*; Figure 5J), accompanied by decreased gene expression. To further validate the DNA methylation status driving these transcriptional alterations, we retrieved the CpG island sequences of these gene promoters from the University of California Santa Cruz (UCSC) Genome Browser database and subsequently performed methylated DNA immunoprecipitation PCR (MeDIP-PCR). The results demonstrated that FoxO3a overexpression significantly enhanced the enrichment of 5-methylcytosine (5-mC) at the promoter regions of *Slc25a4*, *Cox5a*, *Sdhc*, and *Atp6v0c* compared with CON cells (Figure 5K). Moreover, when AML-12 cells, a well-established mouse hepatocyte cell line, were transfected with FoxO3a-overexpressing vectors and subsequently treated with SGI-1027, a potent inhibitor of the DNA methyltransferases DNMT1, DNMT3A, and DNMT3B, the suppressed mRNA levels of *Atp6v0c* and *Slc25a4* were markedly restored (Figure 5L). Collectively, these findings indicate that FoxO3a represses the transcription of target OXPHOS genes in a promoter DNA methylation-dependent manner, thereby establishing an epigenetic silencing mechanism that may play a critical role in the modulation of OXPHOS processes.

### FoxO3a restricts hepatocyte proliferation via CCND1 and promotes apoptosis

**Figure 6:**
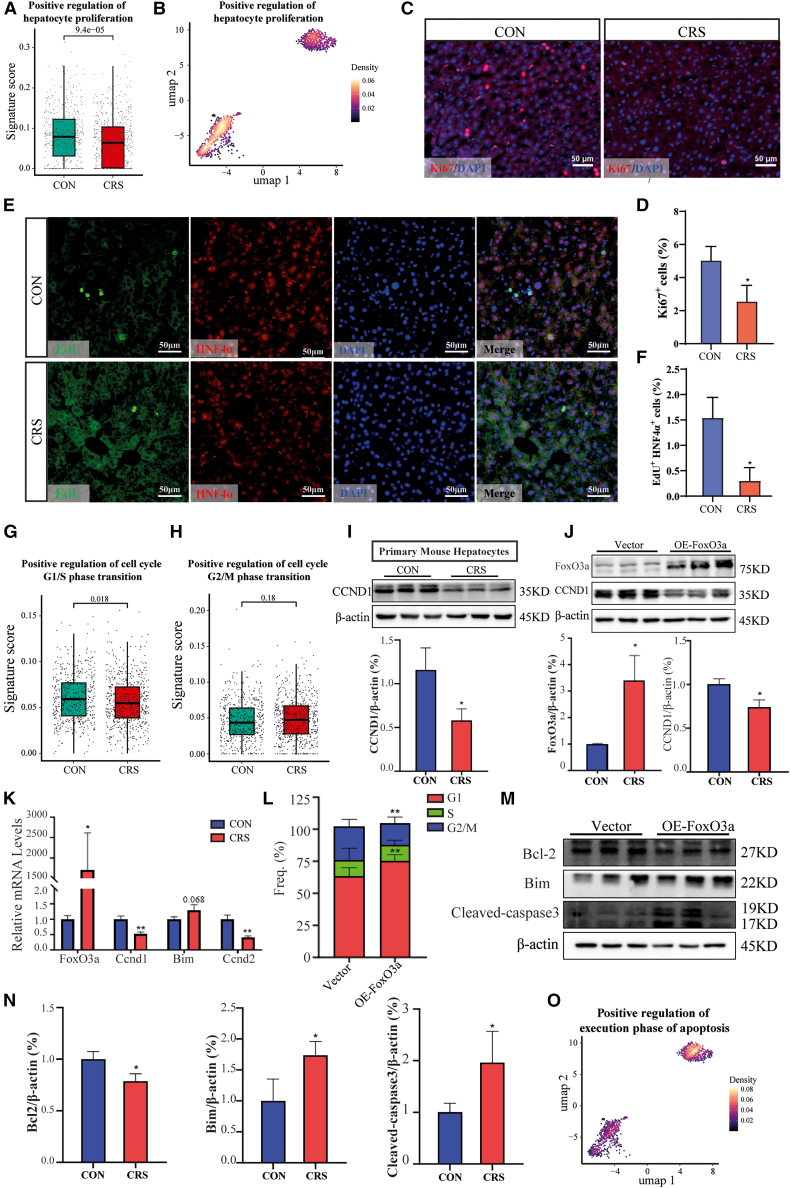
Upregulation of FoxO3a inhibits hepatocyte proliferation (A) Impaired proliferative capacity in CRS hepatocytes. Boxplot comparison of proliferation signature scores between healthy and CRS donors (AUCell quantification). Center line = median; box limits = 25th/75th percentiles; whiskers = 1.5× IQR. Significance was assessed by a two-sided Wilcoxon test. (B) UMAP projection revealing subcluster-specific suppression of proliferation signatures. A purple-to-yellow gradient depicts increased scores. (C) Ki67 staining of liver sections from CON and CRS mice. (D) Analysis of the percentage of Ki67-positive cells. (E and F) Representative images (E) and quantification of EdU^+^ HNF4α^+^ cells (F) in CON and CRS mice. (G and H) Comparison of (G) G1/S phase transition and (H) G2/M phase transition signature scores in hepatocytes from CON and CRS mice. Statistical significance was assessed by a two-sided Wilcoxon test. (I) Western blot analysis of CCND1 in hepatocytes isolated from CON and CRS mice. (J) Western blot analysis of CCND1 in AML-12 cells with FoxO3a overexpression. (K) mRNA levels of Bim and cyclins in AML-12 cells transfected with FoxO3a-overexpressing vectors (*n* = 3 mice/group). (L) Flow cytometry analyses of the cell cycle in hepatocytes. (M and N) Western blot analyses of Bcl-2, Bim and cleaved-caspase3 in AML-12 cells transfected with empty vectors or FoxO3a-overexpressing vectors and quantification of the blots (*n* = 3 mice/group). (O) UMAP visualization of positive regulation of the execution phase of apoptosis signature scores in hepatocyte subclusters. A purple-to-yellow gradient depicts increased scores. All quantification data are presented as mean ± SD. *p* values are calculated using a two-tailed unpaired Student’s t-test, ∗*p* < 0.05, ∗∗*p* < 0.01, ∗∗∗*p* < 0.001.

Further analysis of scRNA-seq data showed significant suppression of hepatocyte proliferation in CRS mice (Figure 6A). Among hepatocyte clusters, cluster 1— characterized by increased FoxO3a regulon activity—exhibited a decreased proliferation enrichment score, whereas scores were higher in clusters 2 and 3 (Figure 6B). To validate the effect of CRS on hepatocyte proliferation, we performed Ki-67 staining on liver sections from both groups. The CRS group showed an approximately 50% reduction in Ki67^+^ cells (Figures 6C and 6D). *In vivo* experiments, mice were intraperitoneally injected with EdU 2 h before liver collection. Consistently, the number of EdU^+^ hepatocytes (EdU^+^ HNF4α^+^) was profoundly decreased in the CRS group (Figures 6E and 6F).

Additionally, CRS induced a significant reduction in signature scores for positive regulators of the G1/S phase transition (Figures 6G and 6H), which was confirmed by downregulated CCND1 in primary hepatocytes from CRS mice (Figure 6I). To further investigate the role of FoxO3a in hepatocyte cell-cycle arrest, we overexpressed *FoxO3a* in AML-12 cells and observed that cyclin (Ccnd1 and Ccnd2) expression was remarkably decreased at both the mRNA and protein levels (Figures 6J and 6K). Flow cytometry analysis showed an increased proportion of cells in the G1 phase and a decreased proportion in the G2/M phase (Figure 6L). Moreover, *FoxO3a* overexpression induced the expression of pro-apoptotic factors, including Bim and cleaved caspase-3, while reducing the anti-apoptotic protein BCL-2 (Figures 6M and 6N). This supports the positive correlation between FoxO3a regulon activity and apoptosis in hepatocyte subclusters (Figures 3K and 6O). Therefore, FoxO3a-mediated dysregulation of cellular processes plays an important role in CRS-induced hepatic injury.

### Inhibition of the PI3K/AKT signaling pathway increases FoxO3a expression

**Figure 7:**
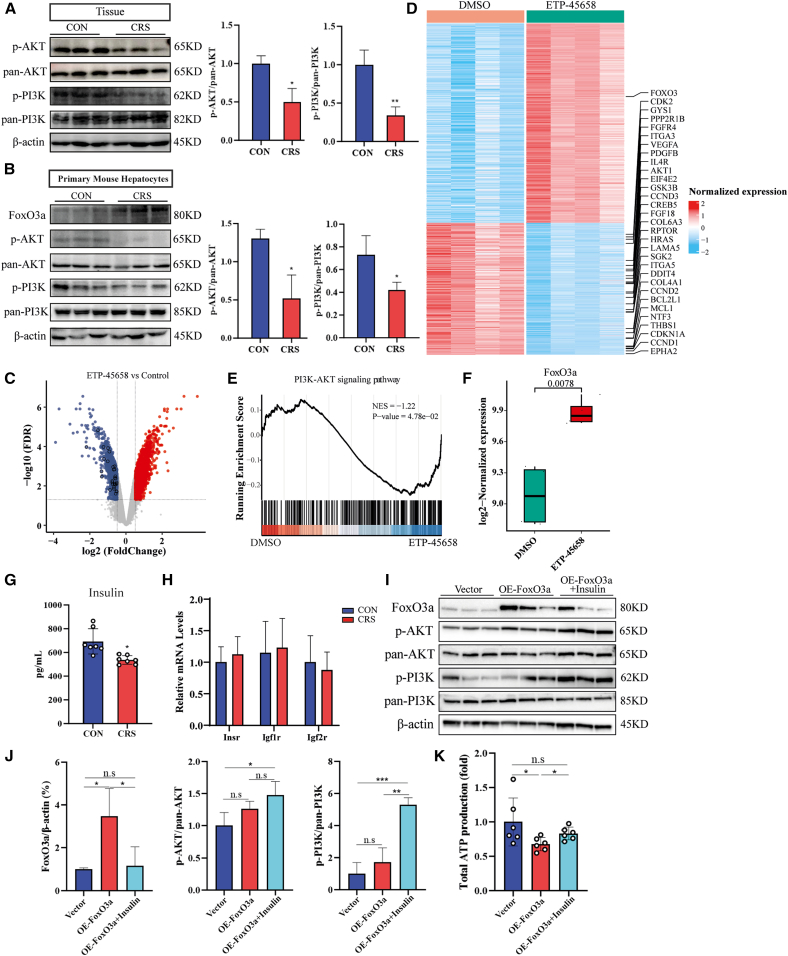
Inhibition of the PI3K/AKT pathway upregulates FoxO3a expression (A and B) Western blot analyses of p-PI3K, PI3K, *p*-AKT and AKT in livers and hepatocytes from CON and CRS mice. Densitometric quantifications are shown accordingly. (C) Volcano plot illustrating transcriptional changes after PI3K inhibition (ETP-45658). Differentially expressed genes (DEGs) were identified using the criteria of false discovery rate (FDR) < 0.05 and |log2 (fold change)| > 0.5. Black circles denote PI3K/AKT pathway-enriched genes. Red and blue denote upregulated and downregulated genes, respectively, after PI3K inhibition. (D) Heatmap showing DEG expression patterns with annotated FoxO3a and core PI3K/AKT genes. A blue-to-red gradient reflects increased expression abundance. (E) Gene set enrichment analysis (GSEA) plot confirming significant suppression of PI3K/AKT signaling following ETP-45658 treatment. (F) Comparative analysis of FoxO3a expression between PI3K inhibition and DMSO control groups. Boxplot elements: center line = median, box limits = first and third quartiles, whiskers = 1.5 × interquartile range (IQR). (G) ELISA of insulin levels in serum from CON and CRS mice (*n* = 7). (H) mRNA levels of InsR, Igf1R, and Igf2R in hepatocytes from CON and CRS mice (*n* = 3 mice/group). (I) Western blot analysis of the PI3K/AKT/FoxO3a axis in AML-12 cells. (J) Quantification of the blots. (K) Measurement of ATP levels in AML-12 cells with insulin treatment. Statistical significance was determined by a two-sided unpaired Student’s *t* test. Data are represented as means ± SD (∗*p* < 0.05, ∗∗*p* < 0.01, ∗∗∗*p* < 0.001).

In neurodegenerative diseases, it has been reported that PI3K/AKT-mediated phosphorylation promotes cytosolic translocation of FoxO3a, thereby suppressing its transcriptional activity.^34^^,^^35^ However, whether this regulatory axis functions in CRS mice remains unclear. In liver tissues and hepatocytes of CRS mice, we observed reduced protein levels of p-PI3K and *p*-AKT (Figures 7A and 7B). We therefore postulated that the PI3K/AKT pathway was inhibited following CRS, thereby increasing the activation of FoxO3a. To assess the inhibitory effect of the PI3K/AKT pathway on FoxO3a, we profiled gene expression in MCF-7 cells treated with PI3K inhibitors (ETP-45658 and PI-103). Compared to the DMSO-treated control, PI3K/AKT inhibition significantly altered downstream gene transcription: ETP-45658 induced upregulation of 1,951 genes and downregulation of 1,286 genes, while PI-103 elicited upregulation of 1,483 genes and downregulation of 955 genes (Figures 7C–7E and S7A–S7C). As expected, FoxO3a expression was significantly upregulated after PI3K/AKT inhibition (Figures 7F and S7D), confirming that the PI3K/AKT pathway negatively regulates FoxO3a expression.

Notably, our previous scRNA-seq analysis revealed significant enrichment of the insulin signaling pathway in CRS mice based on DEGs compared to CON. As a crucial signaling molecule, insulin activates the PI3K/AKT pathway via binding to the insulin receptor (Insr).^36^^,^^37^^,^^38^ Consistently, we observed that serum insulin levels were significantly decreased in CRS mice (Figure 7G), whereas the expression of insulin receptor (IR) and its related receptors (Igf1r/Igf2r) remained unaltered (Figure 7H). This reduction in extracellular insulin stimulation likely contributes to the observed inhibition of the PI3K/AKT axis. To determine whether insulin regulates FoxO3a activity through this pathway, AML-12 cells were treated with insulin after overexpression of FoxO3a. Insulin treatment activated the PI3K/AKT signaling pathway, as evidenced by increased phosphorylation of PI3K (p-PI3K) and AKT (*p*-AKT), which subsequently suppressed FoxO3a expression (Figures 7I and 7J). Furthermore, this insulin-mediated signaling activation restored cellular ATP production (Figure 7K).

## Discussion

In our study, we established a psychological stress mouse model exhibiting impaired liver function, characterized by significant hepatocyte vacuolization, elevated liver transaminase levels, inhibited cell proliferation, and elevated hepatocyte apoptosis. In addition, scRNA-seq data demonstrated that hepatocytes from CRS mice exhibited a decreased capacity for OXPHOS, particularly in periportal hepatocytes of zone 1, consistent with abnormal mitochondrial swelling and rupture. Mechanistically, we found that upregulation of FoxO3a contributed to reduced OXPHOS capacity through FoxO3a-driven epigenetic gene silencing, inhibited hepatocyte proliferation, and enhanced apoptosis. Furthermore, this upregulation of FoxO3a was driven by suppression of the PI3K/Akt signaling pathway, potentially in response to reduced insulin signaling.

Within the hepatic lobule, hepatocytes are conventionally divided into three zones: zone 1 (closest to the portal vein and hepatic artery), zone 3 (near the central vein), and zone 2 (the intermediate region).^8^ In our study of psychological stress-induced liver impairment, we are the first to report that CRS reduces the capacity for OXPHOS in cluster 1 hepatocytes, corresponding to zone 1 periportal hepatocytes. Zone 1 hepatocytes are crucial for gluconeogenesis and beta-oxidation, as this region is relatively rich in oxygen and nutrients due to its proximity to the portal vein.^39^ Moreover, the portal vein is densely innervated by sympathetic nerve axons, whereas sympathetic innervation of the central vein is limited.^40^ This structural difference may contribute to CRS-induced zonally stratified liver dysfunction. Notably, OXPHOS impairment is common across various tissues and cells under stress. In a mouse model of chronic stress, psychological stress disrupts gut homeostasis and lowers the basal level of OCR in intestinal stem cells.^41^ Additionally, skin fibroblasts from patients with major depressive disorder (MDD) exhibit impaired mitochondrial function and respiratory capacity.^42^ We postulate that certain depression-associated pathological molecules may systematically alter mitochondrial function and impair the capacity for OXPHOS in various tissues. Further investigations are needed to elucidate the mechanisms linking psychological stress and OXPHOS.

FoxO3a was identified in this model as a critical regulator of the mechanisms connecting psychological stress to hepatic dysfunction. Our data indicated that CRS-induced metabolic disorder reduced glucose and insulin levels, which in turn downregulated the PI3K/AKT pathway, thereby activating FoxO3a. Upregulation of FoxO3a mediated hepatic injury probably through two mechanisms. First, it suppresses OXPHOS. Our data from CRS mice are the first to demonstrate an inverse correlation between FoxO3a expression and OXPHOS capability. Consistently, hepatocytes from PiZ mice with enhanced FoxO3a activity exhibit lower OXPHOS capability.^33^ Furthermore, we validated that overexpression of FoxO3a reduced OXPHOS capacity in AML-12 cells, whereas its knockdown enhanced this capacity. These findings align with published reports indicating that FOXO3a decreases TCA cycle activity and respiration.^43^ Maintaining optimal OXPHOS is crucial for hepatocytes: as hepatocytes polarize, they require increased cellular energy and TCA activity^44^; similarly, during cell cycle progression and DNA replication, hepatocytes demand more cellular energy.^45^ Consequently, reduced OXPHOS contributes to the disruption of hepatocyte homeostasis by impairing critical cellular processes such as polarization and cell cycle progression. Regarding the mechanism by which FoxO3a regulates OXPHOS capacity, we propose that DNA methylation enzymes may drive the epigenetic gene silencing involved in the OXPHOS process following FoxO3a activation, as evidenced by the upregulation of DNMT3a and the reduced expression levels of OXPHOS-related genes. Second, FoxO3a induces cell-cycle arrest and promotes hepatocyte apoptosis. FoxO3a is typically inactivated in many types of cancer cells and is implicated in regulating cell proliferation, apoptosis, and resistance to oxidative stress.^46^^,^^47^^,^^48^ Restoration of FoxO3a activity has been proposed as a potential therapeutic strategy in cancer treatment. Metformin has been studied to block non-small-cell lung cancer (NSCLC) cells though restoration of FoxO3a,^49^ which results in upregulation of FoxO3a and suppressed breast cancer tumorigenesis.^50^ Collectively, FoxO3a acts as a master regulator mediating the disruption of liver homeostasis during psychological stress.

Our study underscores the pivotal role of FoxO3a in stress-induced liver diseases. These findings are consistent with recent reports by Wang et al., who demonstrated that FoxO signaling is a critical mediator of fluoride-induced hepatotoxicity via the activation of p53-dependent ferroptosis.^51^ Future research should prioritize elucidating the precise mechanisms by which FoxO3a regulates cellular adaptation to chronic stress, with a specific focus on its interplay with key signaling pathways such as PI3K/AKT, and investigating strategies to modulate the PI3K/AKT pathway or other regulators to alleviate liver damage, offering new therapeutic approaches for chronic stress-related liver diseases. In addition, epidemiological studies have consistently highlighted the beneficial effects of emotional well-being on liver health^52^^,^^53^^,^^54^: positive emotional states, such as happiness and contentment, are linked to reduced risks of liver-related and other diseases,^55^ whereas chronic emotional disturbances, such as anger, depression, and anxiety, are associated with increased susceptibility to liver dysfunction and slower recovery from liver diseases.^56^^,^^57^^,^^58^ These findings emphasize the importance of mental health in maintaining liver function and promoting disease recovery.

In our CRS model, we observed a decreased spleen-to-body weight ratio, a finding that aligns with previous reports.^59^^,^^60^ This reduction in spleen size was accompanied by structural disorganization, specifically characterized by white pulp expansion.^59^ The decreased spleen size has been attributed to inhibited cell proliferation^61^ and the release of splenocytes.^59^ Consequently, the splenocyte composition shifted, showing an increased percentage of M2-type macrophages and NK cells.^61^ These stress-induced phenotypes are mediated by activation of the hypothalamic-pituitary-adrenal (HPA) axis and the sympathetic nervous system.^59^^,^^61^ Beyond the metabolic focus of the current study, whether CRS-induced immune alterations involve FoxO3a regulation and subsequent liver injury warrants further investigation to better understand the mechanisms underlying stress-induced hepatic dysfunction.

In summary, our study reinforces the concept that psychological stress negatively impacts liver homeostasis. We characterized CRS-induced liver dysfunction, marked by significant vacuolization in hepatocytes, increased liver transaminase levels, and increased hepatocyte apoptosis. Additionally, our findings demonstrated that CRS-induced reductions in serum insulin levels inhibit the PI3K/AKT pathway, resulting in the activation of FoxO3a, which acts as a master regulator of OXPHOS reduction in periportal hepatocytes, cell cycle inhibition, and apoptosis enhancement. Collectively, these findings suggest that maintaining liver health may be supported through psychosocial intervention and by targeting the FoxO3a signaling pathway.

### Limitations of the study

While this study provides important insights into FoxO3a-mediated liver impairment, several limitations should be acknowledged. First, the sample size in our scRNA-seq analysis is relatively modest. A larger cohort would enhance the generalizability of our findings. To mitigate this limitation, we sought to validate our key findings using molecular experimental approaches, which consistently supported the role of dysregulated FoxO3a in liver damage. Second, the precise mechanism through which chronic stress selectively impairs periportal hepatocytes remains to be fully elucidated. Our data indicate zone-specific vulnerability, but the underlying mechanisms warrant further investigation. Finally, whether the observed pathway—specifically, the PI3K/AKT/FoxO3a-mediated dysregulation of OXPHOS—is conserved in other social stress paradigms, such as chronic unpredictable stress, warrants further investigation. Moreover, whether this mechanism underlies the hepatic consequences of chronic psychological stress in humans remains an open question. Translational studies using human samples will be essential to determine the clinical relevance of our findings and their potential as therapeutic targets.

## Resource availability

### Lead contact

Further information and requests for resources and reagents should be directed to and will be fulfilled by the lead contact, Run Xiao (xiaorun1984@ucas.ac.cn).

### Materials availability

This study did not generate new unique reagents. All materials generated during this study are available from the lead contact upon request.

### Data and code availability

All relevant data and resources can be found within the article and its supplemental information. scRNA-seq data files generated in this study have been deposited in the Genome Sequence Archive (Genomics, Proteomics & Bioinformatics 2025) at the National Genomics Data Center (Nucleic Acids Res 2025), China National Center for Bioinformation/Beijing Institute of Genomics, Chinese Academy of Sciences (GSA: CRA033555, https://ngdc.cncb.ac.cn/gsa/browse/CRA033555), and are publicly accessible.^62^^,^^63^ Other sequencing data analyzed in this study are from existing, publicly available sources. The corresponding links are provided in the key resources table. Algorithms used for data analysis are listed in the key resources table and are publicly available. Any additional information required to reanalyze the data reported in this paper is available from the lead contact upon request.

## Acknowledgments

The authors acknowledge the support from the Shared Instrumentation Core Facility at the Hangzhou Institute of Medicine (HIM), 10.13039/501100002367Chinese Academy of Sciences. This work was supported by The Zhejiang Provincial Natural Science Foundation of China under grant no. YXD23H0303, the Key R&D Program of Zhejiang (2024C03208), and research grants (2021QD01 and 2025ZZBS10) from the Hangzhou Institute of Medicine, Chinese Academy of Sciences. Additional support was provided by The 10.13039/501100001809National Natural Science Foundation of China (82270193 and 82470156) and The 10.13039/501100004731Zhejiang Provincial Natural Science Foundation of China (YXD24H0801).

## Author contributions

Conception and design, Y.W., R.Z., and L.Z.; methodology, Y.W., R.Z., M.L., Z. Yuan, H.Q., and X.N.; investigation, W.C. and B.W.; writing original draft, Y.W., R.Z., and R.X.; review & editing, H.I., R.Z., U.U.R., and R.X.; visualization, Y.W., R.Z., Z.J., and Z. Yi; study supervision, L.L., H.L., and R.X.; funding acquisition, R.X.

## Declaration of interests

The authors declare no competing interests.

## STAR★Methods

### Key resources table

**Table:** 

REAGENT or RESOURCE	SOURCE	IDENTIFIER
**Antibodies**		

rabbit anti-Actin	Proteintech	Cat#AC026
mouse anti-GAPDH	Proteintech	Cat# 60004-1-lg
rabbit anti-FKHRL1	Santa Cruz	Cat# sc-48348; RRID: AB_627634
rabbit anti-HNF4α	ABclonal	Cat# ab201460
rabbit anti-Cyclin D1	ABclonal	Cat# A19038; RRID: AB_2862530
rabbit anti-AKT	ABclonal	Cat# A18675; RRID: AB_2862411
rabbit anti-*p*-AKT(Ser473)	ABclonal	Cat# AP1453
rabbit anti-PI3K	ABclonal	Cat# A22996
rabbit anti-p-PI3K	ABclonal	Cat# AP1463
rabbit anti-Bim	ABclonal	Cat# A19702; RRID: AB_2862744
rabbit anti-Bcl2	CST	Cat# 3498T; RRID: AB_1903907
rabbit ant-cleaved-Caspase3	CST	Cat# 14220T; RRID: AB_2798429
rabbit anti-Ki67	ABclonal	Cat# A26419PM
goat anti-mouse HRP	Bio-rad	Cat#1706516; RRID: AB_2921252
goat anti-rabbit HRP	Bio-rad	Cat#1706515; RRID: AB_11125142
Alexa Fluor 488 goat Anti-Rabbit IgG(H + L)	Abcam	Cat# ab150077; RRID: AB_2630356
Alexa Fluor 555 goat Anti-Rabbit IgG(H + L)	Abcam	Cat# ab150078; RRID: AB_2722519

**Bacterial and virus strains**		

DH5α Chemically Competent Cell	Beyotime	N/A
Stbl Chemically Competent Cell	Beyotime	N/A
rAAV8-shRNA-FoxO3A	Angel Biotech	N/A
rAAV8-shRNA	Angel Biotech	N/A

**Chemicals, peptides, and recombinant proteins**		

FBS	Noverse	Cat# NFBS-2500A
RIPA	Solarbo	Cat# R0020
Protease inhibitor	Yeasen	Cat# 20124ES03
phosphatase inhibitor	Yeasen	Cat# 20109ES05
penicillin-streptomycin	Biosharp	Cat# BL505A
Triton X-100	Biofoxx	Cat# 1139ML500
BSA	Biofoxx	Cat# 4240GR100
DAPI	Beyotime	Cat# C1002
Insulin	MCE	Cat# HY-P0035
SGI-1027	MCE	Cat# HY-13962

**Critical commercial assays**		

BCA Protein Assay Kit	Beyotime	Cat# P0012
TUNEL Kit	Beyotime	Cat# C1086
HiScript IV ALL-in-One Ultra RT SuperMix	Vazyme	Cat# R333-01
EndoFree Plasmid Midi Kit	TIANGEN	Cat# DP117
qPCR SYBR Green Master Mix(No Rox)	Yeasen	Cat# 11201ES08

**Deposited data**		

scRNA sequence data	This paper	https://ngdc.cncb.ac.cn/gsa/search?searchTerm=CRA033555
RNA-seq (PiZ mouse livers)	Piccolo et al.^33^	https://doi.org/10.1073/pnas.2025242118
RNA-seq (PI3K inhibition)	Hill et al.^64^	https://doi.org/10.1186/s13058-014-0482-y
ATAC-seq and RNA-seq data(FoxO3a-induced mESCs)	Santini et al.^32^	https://doi.org/10.1038/s41467-024-51794-9
XF Cell Mito Stress Test Kit	Agilent	Cat# 103015-100

**Experimental models: Cell lines**		

AML-12	Mingzhou	Cat# MZ-0921

**Experimental models: Organisms/strains**		

Mouse: C57BL/6J	GemPharmatech	N/A

**Oligonucleotides**		

the List of primer	Sangon Biotech	Table S1
FoxO3a regulon	Sangon Biotech	Table S2

**Software and algorithms**		

GraphPad Prism 10 (v10.1.2)	GraphPad	https://www.graphpad.com/
WPS Excel software	WPS	https://www.wps.cn/
Flowjo	BD Bioscience	https://www.flowjo.com/
ImageJ software	ImageJ	https://imagej.net/software/fiji/
CeleScope	Singleron	https://github.com/singleron-RD/CeleScope
STAR	Dobin et al.^65^	https://github.com/alexdobin/STAR
FeatureCounts	Liao et al.^66^	https://github.com/byee4/featureCounts/tree/master
Seurat	Hao et al.^67^	https://satijalab.org/seurat/
AUCell	Aibar et al.^68^	https://github.com/aertslab/AUCell
Harmony	Korsunsky et al.^69^	https://github.com/immunogenomics/harmony
GSVA	Hänzelmann et al.^70^	https://github.com/rcastelo/GSVA
pySCENIC	Van et al.^71^	https://github.com/aertslab/pySCENIC
Bowtie2	Langmead et al.^72^	https://bowtie-bio.sourceforge.net/bowtie2/index.shtml
HISAT2	Kim et al.^73^	https://daehwankimlab.github.io/hisat2/
Samtools	Danecek et al.^74^	https://github.com/samtools/samtools
Picard	Broad Institute	https://github.com/broadinstitute/picard
deepTools	Ramírez et al.^75^	https://github.com/deeptools/deepTools
IGV	Thorvaldsdóttir et al.^76^	https://github.com/igvteam/igv
DESeq2	Love et al.^77^	https://github.com/thelovelab/DESeq2
clusterProfiler	Wu et al.^78^	https://github.com/YuLab-SMU/clusterProfiler

### Experimental model and study participant details

#### Animals

C57BL/6J female mice (4 weeks) were housed in cages with a 12-h light/dark cycle and with food and water *ad libitum*. The research complied with the Institutional Animal Care and Use Committee guidelines and was approved by the Animal Care Committee of Tianjin University (Approval Number: AP2024-07-004). All mice were aged 4 weeks at the start of experiments.

#### Cell lines

Alpha mouse liver 12 cell lines AML-12 were bought from the Mingzhou Bio. All the cell lines were authenticated by short tandem repeat (STR) profiling and tested for mycoplasma contamination. For drug treatment, cells were seeded at 1 × 10^5^ per well, transfected with 4 μg of plasmids per well, and subsequently treated with 10 μM SGI-1027 (HY-13962, MCE) or Insulin (HY-P0035, MCE) for 24 h.

### Method details

#### Chronic restraint stress procedures

Female 4-week-old mice were housed in groups (5 mice per cage) in cages. After 5 days of acclimatization to the environment, mice were subjected to restraint stress with 50-mL centrifuge tubes with sufficient holes punched on them to allow them to breathe and to prevent an acute rise in body temperature.^79^ They were restrained daily for 6 h, from 11:00 a.m. to 5:00 p.m., for 21 consecutive days. For AAV administration, mice received a single tail vein injection of AAV (1×10^11^ vg per animal) at 4 weeks of age, followed by a 4-week monitoring period and a subsequent 3-week chronic restraint stress prior to sacrifice at the experimental endpoints.

#### Animal behavior test

Elevated Plus Maze Test: The elevated plus maze consisted of two open and two closed arms. Mice activities were tracked for 5 min with a video tracking system (Noldus EthoVisionXT). The time spent in the open arms was measured.

Open Field Test: Mice were put into the center of an open field box and their activities were recorded for 5 min with a video tracking system (Noldus EthoVisionXT). The time spent in the center zone and border zone, as well as the distances traveled was measured.

Tail Suspension Test: Mice were suspended from a tail suspension frame and their behaviors were recorded for 6 min by a video tracking system (Noldus EthoVisionXT). The time spent immobile was measured.

#### H&E histology

The liver tissues were embedded in paraffin after being fixed for at least 12 h. Sections were stained with hematoxylin and eosin (H&E).

#### Flow cytometry

AML-12 cells were rinsed with PBS and digested from the 6-well plate by trypsin. Centrifuge cells at 4°C, 1000 rpm for 3 min. Resuspend with 300 μL PBS followed by adding 700 μL 100% ethanol for fixation. Incubate at 4 °C overnight and wash with PBS. Add 1 mL of 20 μg/mL RNase solution to the resuspended cells for 1 h and add 3 μL Propidium Iodide (1 mg/mL) to the cell suspension for 20 min. After staining, single cell suspensions were analyzed by CytoFLEX LX.

#### Isolation of primary mouse hepatocytes

The anesthetized mice livers were perfused via the portal vein with 20 mL D-Hank’s containing 5 mM EDTA (pH 8.0), followed by pre-warmed 20 mL DMEM/F12 (Gibco) containing 100 U/mL collagenase IV (Gibco). The livers were dissected gently and placed in ice-cold DMEM/F12. The Glisson’s capsule was then ruptured to release the cells into the medium, which were filtered through a 100 μm cell strainer. The cell suspension was centrifuged at 100 g for 5 min at 4 °C, and the pellet was resuspended with 5 mL ice-cold DMAEM/F12. The cell suspension was added to 5 mL 40% percoll (Biosharp) slowly and spin at 400 g for 10 min at 4 °C (lowest acceleration and brake). The hepatocytes pellet was resuspended with DMEM containing 10% FBS, 100 U/mL penicillin and 100 mg/mL streptomycin. The hepatocytes density was adjusted to 6 × 10^5^ cells/mL and seeded into Agilent Seahorse XF24 microplate (100 μL per well). An additional 150 μL of medium was added after culturing at 37°C for 6 h.

#### Immunofluorescence

Mouse were perfused with 0.1 M phosphate-bufffered saline (PBS) followed by 4% paraformaldehyde (PFA). The livers were removed and post-fixed overnight in 4% PFA and then dehydration with 30% sucrose. Mouse liver sections were incubated in blocking solution for1 h at room temperature after washing by PBS for three times.

Primary antibodies were applied for 1 h at room temperature after washing by PBS. Secondary antibodies were incubated for 1 h at room temperature. Images were acquired a using confocal microscope (Nikon, A1 HD25).

#### TUNEL assay

The apoptotic cells in the liver sections of mice were detected by One Step TUNEL Apoptosis Kit (Cat# C1086, Beyotime, Shanghai, China). Images were obtained using a confocal microscope (Nikon, A1 HD25).

#### Electron microscopy

Mice were perfused with PBS and 4% PFA. Then the livers were dissected and fixed immediately in 2.5% glutaraldehyde at 4°C overnight. The ultrathin sections were observed under TEM (JEM-2100plus). The area and the number of mitochondria were analyzed by ImageJ software.

#### Serum biochemical analysis

Blood samples were collected from the mice and allowed to clot for 1 h at room temperature. Serum was separated by centrifugation at 4°C. Serum concentrations of alanine aminotransferase (ALT), aspartate aminotransferase (AST), alkaline phosphatase (ALP), total bilirubin (T-BIL) were measured by biochemical kits (MedicalSystem) using automatic biochemical analyzer (HITACHI 7180 AUTOMATIC ANALYZER).

#### Real-time quantitative PCR

Total RNA was extracted using TRIzol reagent (Vazyme). cDNA was generated from 2 μg total RNA using FastKing-RT SuperMix (TIANGEN, Cat# KR118-02) according to the manufacturer’s instructions. qPCR was performed using Hieff qPCR SYBR Green Master Mix (Yeasen, Cat# 11201ES08). Primers (all for mouse genes) are listed in Table S1.

#### Western blotting

Livers ang cells were lysed in RIPA buffer (Solaribio, Cat# R0020), supplemented with protease inhibitor (Beyotime, Cat# P1005-1) and phosphatase inhibitor cocktail I (GLPBIO, Cat# GK10011) on ice for 30 min, followed by centrifugation at 12000 rpm for 10 min at 4°C. Protein concentrations were measured using Enhanced BCA Protein Assay Kit (Cat# P0010). Protein was loaded by SDS-PAGE gel and transferred onto PVDF membranes (Millipore), membranes were blocked in 3% BSA for 1 and incubated with primary antibody overnight at 4°C. Membranes were washed with TBST for three times and incubated with secondary antibodies at room temperature for 1 h. Bands were detected by ECL kit (Vazyme, Cat# E411-04). Primary antibodies are shown in key resources table.

#### Cell metabolism (Seahorse) analysis

Primary mouse hepatocytes were seeded at 60,000 cells/well in XF24 cell culture microplates (Seahorse Biosciences, CA, USA) overnight. The cellular OCR was measured by XF Cell Mito Stress Test Kit (Agilent technology) in Seahorse XF24 analyzer (Agilent technology). During the measurement, hepatocytes were exposed to 1 μM oligomycin, 0.5 μM FCCP and 1 μM rotenone/antimycin at indicated time points.

#### *In vivo* BrdU proliferation assay

C57BL/6 mice were injected intraperitoneally with 1 mg/mL solution of EdU (GLPBIO, GC42504) in PBS. After 2 h, livers were collected and fixed in 4% parapormaldehyde (4% PFA) followed by embedded in OCT (Sakura, USA) for frozen section. The EdU+ cells of slice were detected by BeyoClick EdU Cell Proliferation Kit. Hepatocyte proliferation (HNF4α^+^ cells) was assessed by calculating the percentage of proliferating hepatocytes (EdU^+^ HNF4α^+^ cells) relative to the total hepatocyte population (HNF4α^+^ cells).

#### MeDIP assay

The MeDIP procedure was performed as previously described with minor modifications.^80^^,^^81^ Briefly, Genomic DNA was isolated from FoxO3a-overexpressing and control cells using the xxxx kit, according to the manufacturer’s instructions. The extracted DNA was fragmented via sonication using a non-focused ultrasonic homogenizer (Qsonica Q800R3) to generate fragments ranging from 200 to 500 bp. Then, 1 μg of sonicated DNA was heat-denatured and incubated overnight at 4°C with a monoclonal antibody specific for 5-methylcytidine (5-mC; proteintech, cat#68301-1) in immunoprecipitation buffer. Antibody-bound DNA fragments were then captured using Protein A/G magnetic beads, followed by extensive washing. After elution and purification, the immunoprecipitated DNA and input DNA were analyzed by quantitative real-time PCR (qPCR) using gene-specific primers spanning the promoter regions of Slc25a4, Cox5a, Sdhc, and Atp6v0c. The relative enrichment of methylated DNA at each target region was calculated as a percentage of the input DNA.

### Quantification and statistical analysis

#### Single-cell RNA-seq analysis

The fresh liver tissues from CON and CRS mice were immediately stored in the sCelLiveTM Tissue Preservation Solution (Singleron) on ice after the mice were sacrificed. The samples were washed with Hank’s Balanced Salt Solution (HBSS) for three times, cut into small pieces, and then digested in 3 mL sCelLiveTM Tissue Dissociation Solution (Singleron) by Singleron PythoNTM Tissue Dissociation System at 37 °C for 15 min. The cell suspension was collected and filtered through a 40-μm cell strainer. Then, red blood cell lysis (RCLB, Singleron) was added and incubated with suspension at room temperature for 5-8 min to remove red blood cells. Finally, the samples were stained with Trypan Blue to evaluate the cell viability. The scRNA-seq libraries were constructed according to the protocol of the GEXSCOPE Single Cell RNA Library Kits (Singleron) (Ref). Individual libraries were diluted to 4 nM, pooled, and sequenced on Illumina novaseq 6000 with 150 bp paired-end reads.

Raw sequencing reads were processed to generate gene expression profiles using CeleScope v1.12.0 (Singleron Biotechnologies) with default settings. In brief, cell barcodes and UMIs were extracted from the R1 reads. Adapter sequences and poly(A) tails were trimmed from the R2 reads, followed by alignment of the trimmed R2 reads to the GRCm38 using STAR v2.6.1b. Uniquely mapped reads were then assigned to specific genes using FeatureCounts v2.0.1. Finally, reads that were successfully assigned with matching cell barcodes, UMIs, and gene identifiers were aggregated to generate the gene expression matrix. This matrix was further analyzed using the Seurat R package (v2.0.1).^67^

#### Identification of major cell clusters

For each sample dataset, the expression matrix was filtered based on the following criteria: 1) cells with fewer than 200 detected genes or those with gene counts exceeding the top 2% threshold were excluded.; 2) cells with exceeding the top 2% UMI counts were excluded; 3) cells with over 20% UMIs originating from the mitochondrial genome were excluded; 4) genes expressed in fewer than five cells were removed. Cell doublets were identified based on the expression patterns of canonical cell type-specific markers. Clusters showing enrichment for multiple cell type-specific markers were excluded from further analysis. The transcriptome expression matrices of the remaining 23,356 high-quality cells were normalized to the total cellular UMI count and scaled, with the cell cycle score regressed out. To reduce the dimensionality of the dataset, 2000 highly variable genes (HVGs) were selected for principal component analysis (PCA). Cell integration was performed using the Harmony R package.^69^ The top 20 principal components (PCs), selected based on their standard deviations, were subsequently used for UMAP analysis.

#### Heterogeneity analysis of hepatocytes

To assess the heterogeneity of hepatocytes and evaluate the impact of CRS, we isolated cells belonging to the cluster and performed dimensionality reduction using PCA based on HVGs. Subsequently, we applied a graph-based clustering approach utilizing the FindClusters function from the Seurat package. A conservative resolution of 0.3 was selected, with all other parameters set to their default values. The hepatocytes were further reclustered and visualized in UMAP plots.

#### Marker gene identification and cluster annotation

To identify marker genes specific to each cell cluster, we performed pairwise comparisons between cells within a cluster and all other cells using the FindAllMarkers function in the Seurat package, with the parameters set as min.pct = 0.25 and logfc.threshold = 0.25. The resulting cell clusters in the two-dimensional representation were annotated to known biological cell types by integrating the identified marker genes with canonical marker genes (Figure S2). A similar analytical strategy was employed for hepatocyte subclusters. Moreover, the top 10 marker genes for each cluster were visualized in a heatmap.

#### Pathway enrichment and FoxO3a regulon activity analysis

Pathway datasets were obtained from the Molecular Signatures Database (MSigDB).^82^ To evaluate the differential activities of pathways between distinct cell sets (e.g., cells derived from CRS or healthy hepatic tissues, or hepatocyte subclusters), we conducted gene set variation analysis (GSVA) on individual cells using default parameters.

In accordance with the pySCENIC workflow,^71^ we constructed the FoxO3a regulon (Table S2). Briefly, coexpression modules were initially inferred using GRNBoost2 to identify genes coexpressed with FoxO3 from the scRNA-seq gene expression matrix, which highlighted potential regulatory interactions. Subsequently, direct targets were refined by processing these coexpression modules with cisTarget (https://resources.aertslab.org/cistarget/). This step employed enrichment analysis of FoxO3a-specific binding motifs in *cis*-regulatory regions near transcription start sites, pruning indirect targets and retaining only motif-enriched genes. The refined set, consisting of FoxO3a and its direct targets, was defined as the FoxO3a regulon.

Finally, regulon activity was quantified using the AUCell algorithm,^68^ which calculated an enrichment score (area under the curve) based on the recovery of the regulon’s target genes within the ranked gene expression list for each cell. The FoxO3a regulon activity scores were then compared between the predefined cell sets described earlier.

#### Bulk RNA-seq and ATAC-seq analysis of FoxO3a activation effects

To investigate the effects of FoxO3a activation on hepatocytes, we compared bulk RNA-seq data from PiZ mouse livers characterized by increased nuclear accumulation of FoxO3a to wild-type controls at two distinct time points: 6 weeks and 36 weeks.^33^ Differentially expressed genes (DEGs) were identified based on the criteria of FDR <0.05 and |log2FC| > 0.5. Pathways that were activated or suppressed were further analyzed for enrichment using the GSEA function in the clusterProfiler R package.^78^

To validate the regulatory relationship between the PI3K-AKT pathway and FoxO3a, we examined gene expression profiles in MCF7 cells at 6 h post incubation with either ETP-45658 or PI-103 (PI3K inhibitors).^64^ Specifically, we assessed the enrichment of the PI3K-AKT pathway and the expression levels of FoxO3a to confirm its regulatory role.

To assess the impact of FoxO3a activation on chromatin accessibility, we analyzed ATAC-seq and RNA-seq data from mouse embryonic stem cells (mESCs) following FoxO3a induction.^32^ ATAC-seq reads were aligned to the mouse reference genome (mm10) using Bowtie2 (v2.5.1) in end-to-end mode with the parameters “--very-sensitive -X 2000” and subsequently sorted with Samtools (v1.6). PCR duplicates were marked and removed using the Picard toolkit (v2.27.4; https://broadinstitute.github.io/picard/). Peak calling was performed with MACS2 (v2.2.9.1) under default settings, using the options “--nomodel --shift −100 --extsize 200”. Chromatin accessibility signals were normalized to reads per kilobase per million mapped reads (RPKM) with deepTools bamCoverage (v3.5.4)^75^ to enable cross-sample comparison and were visualized using the Integrative Genomics Viewer (IGV, v2.13.2).^76^

RNA-seq reads were aligned to the mouse reference genome (mm10) using HISAT2 (v2.2.1)^73^ with the parameter “--rna-strandness F” to account for strand specificity. Gene expression quantification was performed using featureCounts (v2.0.3) with the option “-s 1” to reflect stranded library preparation. Differentially expressed genes (DEGs) were identified as described previously. To visualize the data in the Integrative Genomics Viewer (IGV, v2.13.2),^76^ raw BAM files were converted to BigWig format using bamCoverage (v3.5.4)^75^ with normalization by RPKM (“--normalizeUsing RPKM”).

#### Statistical analysis

Data were statistically analyzed using GraphPad Prism 10.0 and WPS Excel software. Fluorescence images (fluorescence intensity and cell count) and Western blot images (gray intensity values) were analyzed using ImageJ software. Data were presented as mean ± SD (standard error of the mean). For comparisons between two groups, a two-tailed unpaired *t* test was used, significance levels are indicated as ∗*p* < 0.05, ∗∗*p* < 0.01, and ∗∗∗*p* < 0.001.
