# Supplementary material for: Chronic psychological stress disrupts liver homeostasis by dysregulating oxidative phosphorylation via the PI3K/AKT/FoxO3a axis
Source: iScience. 2026 Mar 18;29(4):115389. doi: 10.1016/j.isci.2026.115389 (PMC13068581; doi:10.1016/j.isci.2026.115389)
Supplement: Document S1. Figures S1–S7 and Tables S1 and S2 [file mmc1.pdf]

iScience, Volume *29*

Supplemental information

Chronic psychological stress disrupts liver homeostasis by dysregulating oxidative phosphorylation via the PI3K/AKT/FoxO3a axis

Yue Wang, Rongjie Zhao, Ziyin Yuan, Lina Zhai, Xin Ning, Man Lv, Zihui Jin, Haroon Iqbal, Uzair Ur-Rehman, Zhou Yi, Wangkai Chen, Baichuan Wang, Haoyue Qianjiang, Lihong Li, Huacheng Luo, and Run Xiao

Figure S1

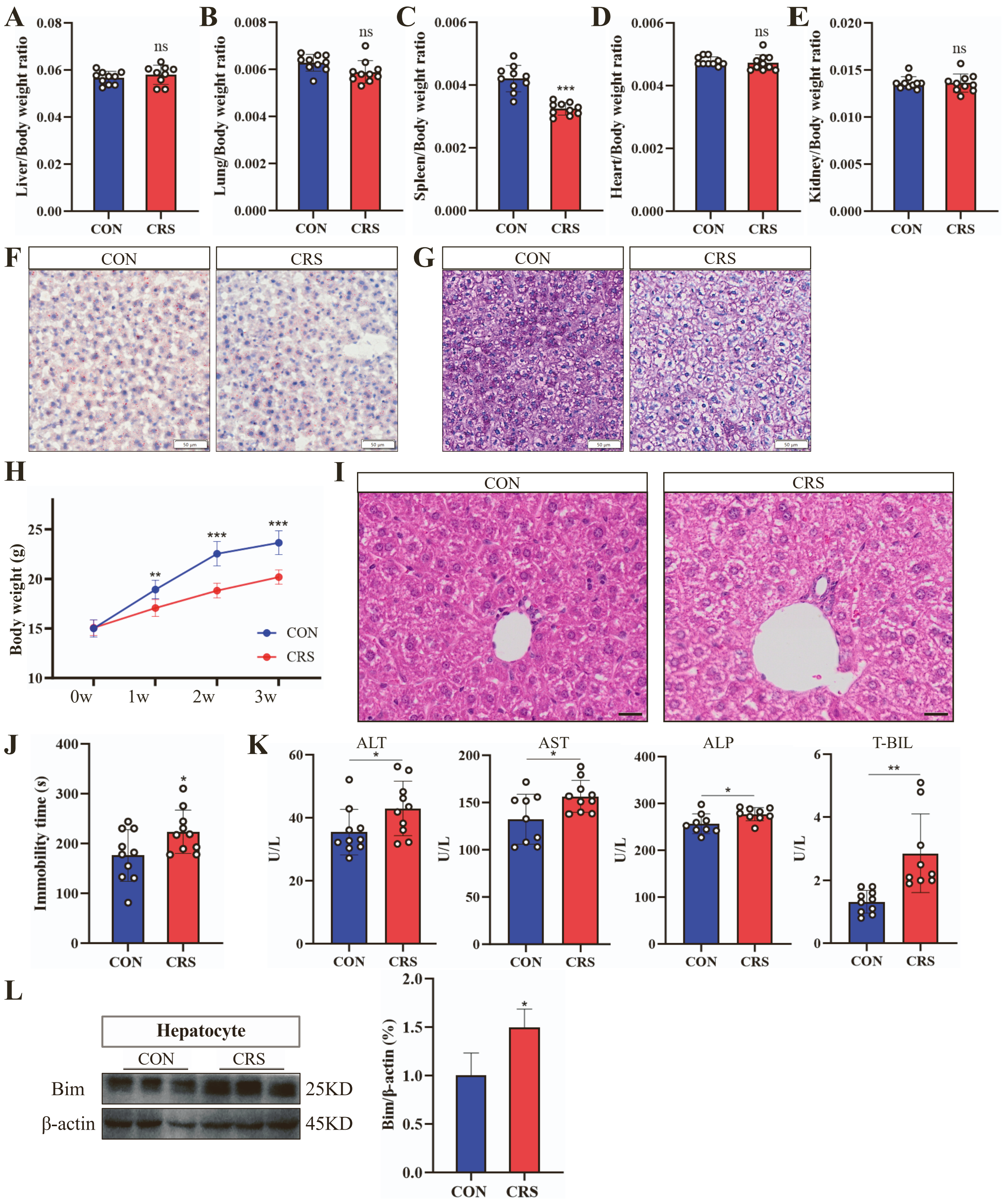

Figure S1. The hepatocyte vacuolation was not caused by the deposition of lipids or glycogen. (A-E) The changes of liver/body weight ratio (A), of lung/body weight ratio (B), spleen/body weight ratio (C), heart/body weight ratio (D) and kidney/body weight ratio (E) (n=10 mice/group). (F) Oil red O staining of liver tissues. (G) PAS staining showed less glycogen deposition in CRS group. (H) Body weight changes of male mice during CRS induction. (I) H&E staining of liver slices in CON and CRS male mice. Scale bar = 50 μm. (J) The immobility time of CON and CRS male mice in TST test (n=10/group). (K) Measurement of serum levels of hepatic function markers: ALT, AST, ALP, T-BIL. (L) Western blotting analyses against Bim in hepatocytes from CON and CRS mice.Data are expressed as means ± SD. The statisticalsignificance of differences was determined unpaired Student's t-test, *$p < 0.05$, **$p < 0.01$, ***$p < 0.001$.

Figure S2

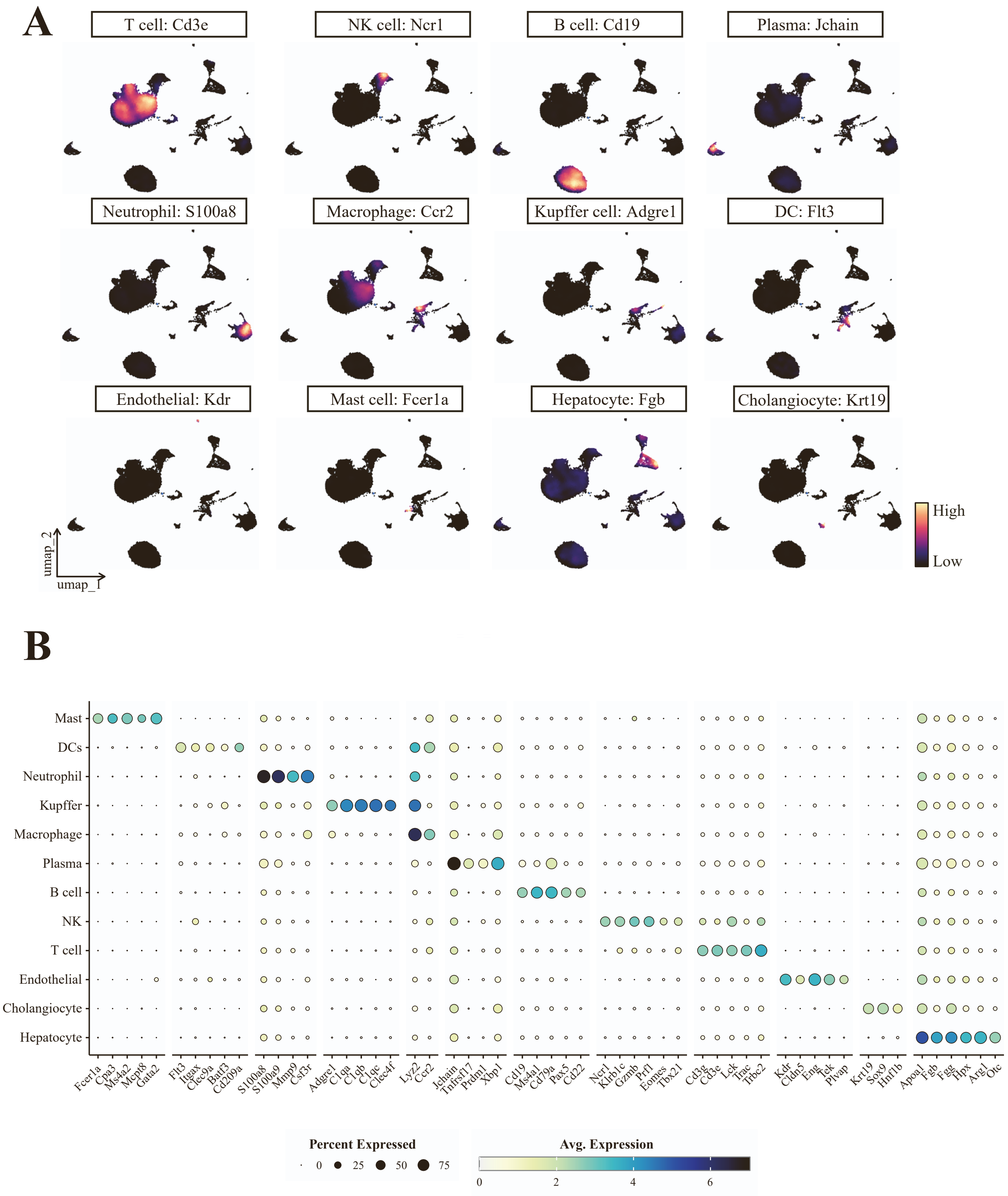

Figure S2. Cell type-specific gene expression patterns. (A) Expression intensity of representative markers is shown in UMAP plot. Purple to yellow continuum depicts increased expression abundance. (B) Dot plot visualization of normalized expression levels across annotated cell clusters. Dot size corresponds to detection frequency (percentage of cells expressing target genes within cluster), while color intensity represents mean expression.

Figure S3

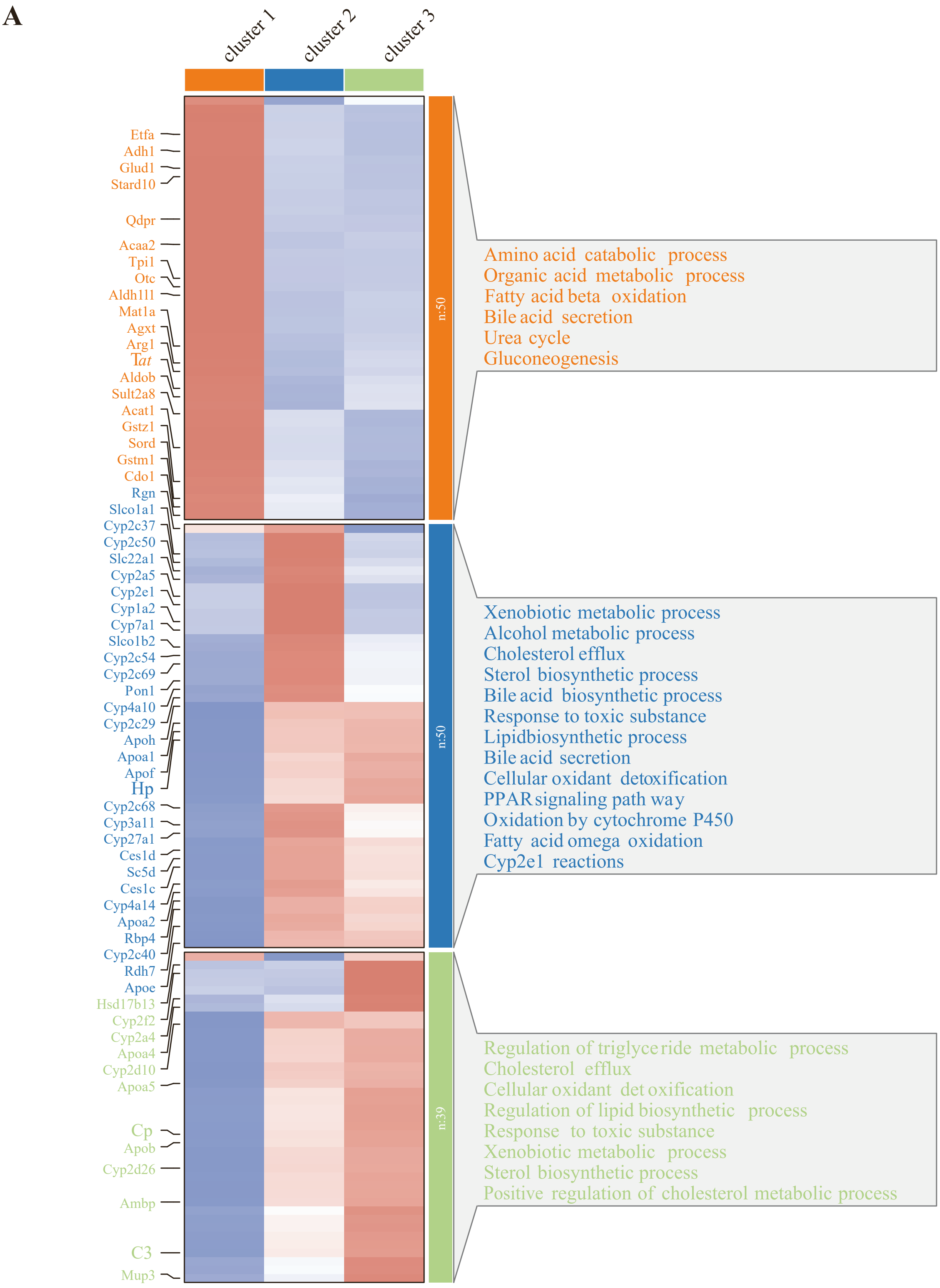

Figure S3. Expression profiles of marker genes specific to hepatocyte subclusters. (A) Z-score normalized heatmap of top 50 marker genes across hepatocyte subclusters and marker genes-involved pathways.

Figure S4

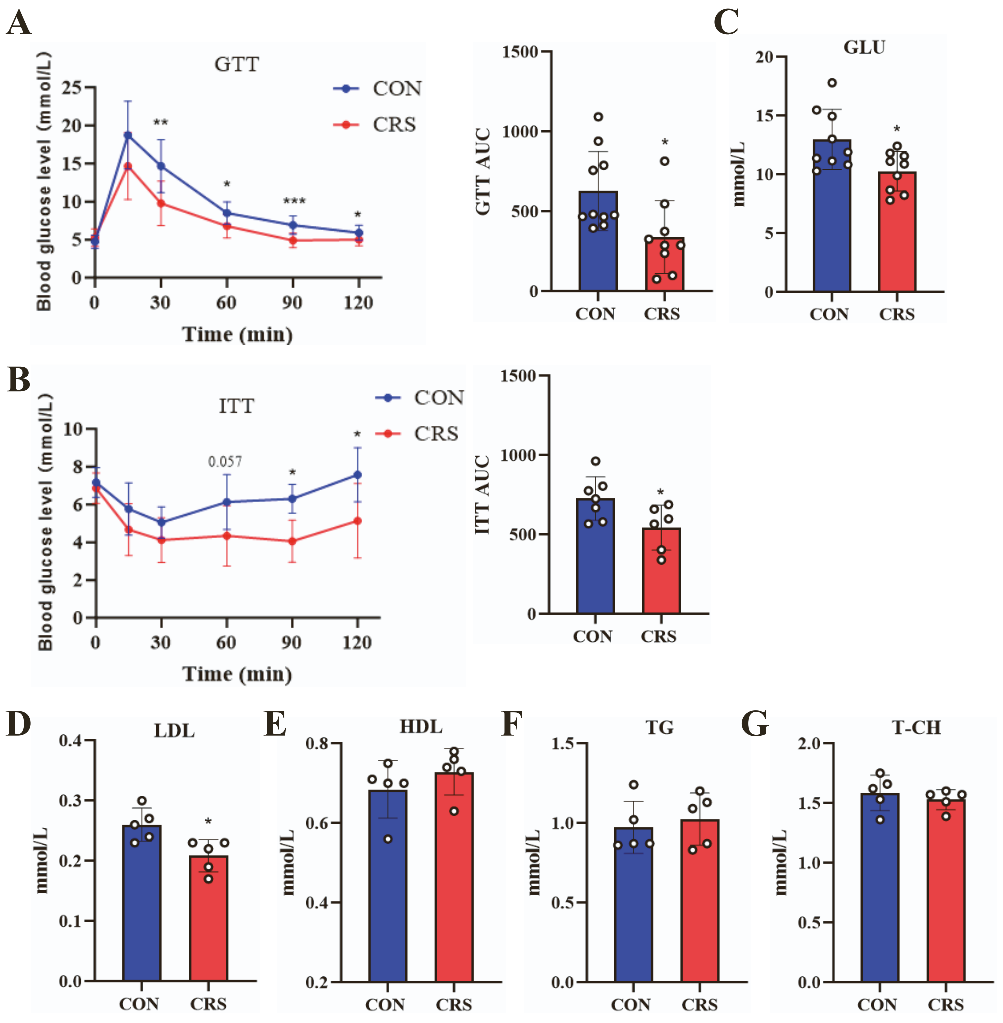

Figure S4. CRS leads to abnormal glucose metabolism in mice. (A-B) Glucose tolerance test (GTT) (A) and insulin tolerance test (ITT) (B) were performed to measure blood glucose at indicated time points (n=10 mice/group). Quantification of the area under curve of GTT and ITT assays were presented at the right panel. (C) Baseline blood glucose levels in mice (n=9 mice/group). (D-G) Quantification oflipid metabolism marker in mouse serum (n=5 mice/group). LDL, low-density lipoprotein, HDL, high-density lipoprotein,TG: triglycerides, T-CH: total cholesterol.Data are represented as means ± SD. The statistical significance of differences was determined by unpaired Student's t-test, *$p < 0.05$, **$p < 0.01$, ***$p < 0.001$.

Figure S5

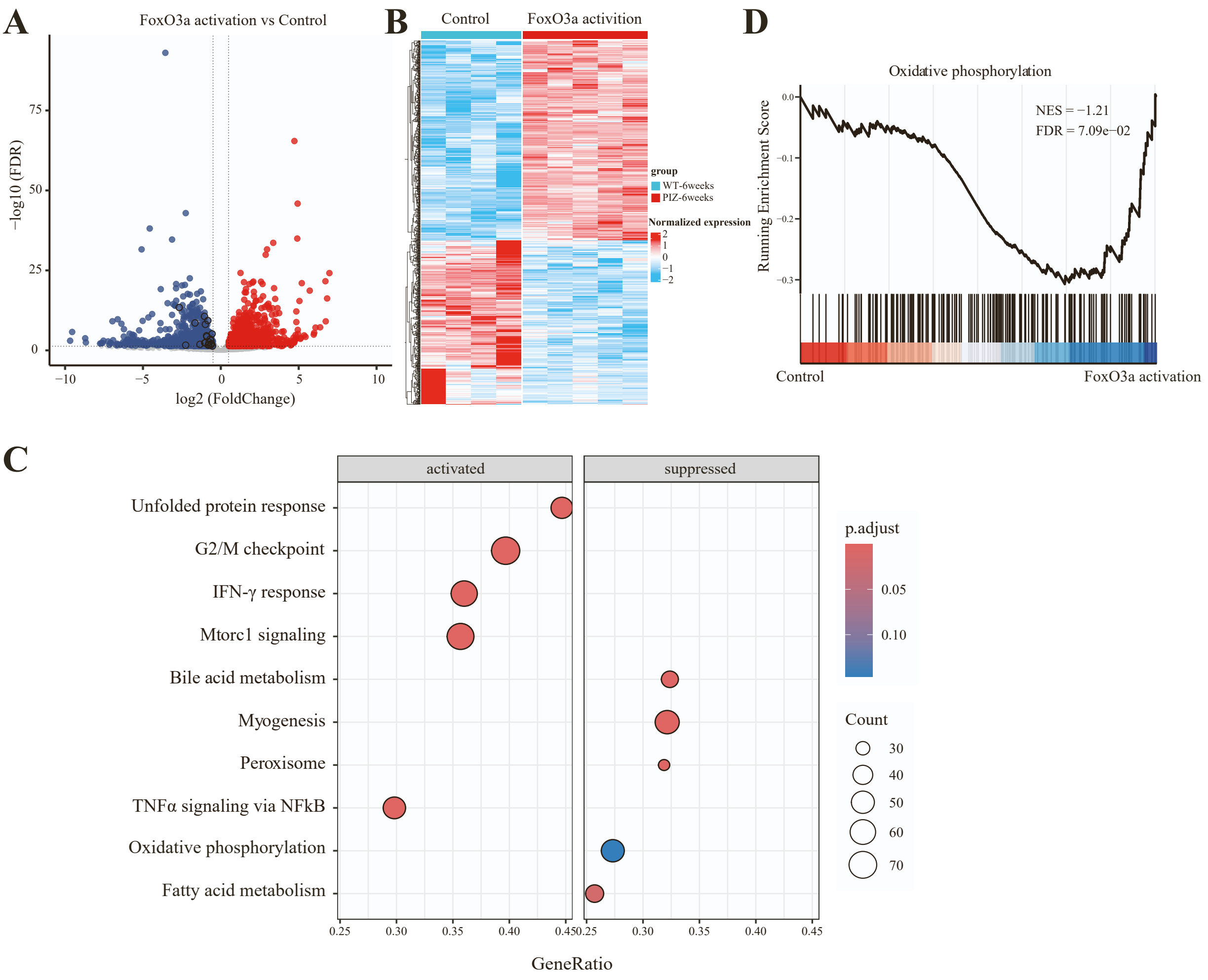

Figure S5. Highly activated FoxO3a impacts hepatic signaling pathway (6-week cohort). (A) Volcano plot of hepatic transcriptional changes induced by FoxO3a activation.Differentially expressed genes (DEGs) between FoxO3a-activated and wild-type murine hepatic tissues were identified (FDR < 0.05, |log2FC| > 0.5). Red/blue denote up-/down-regulated genes in FoxO3a-activated tissue. (B) Heatmap reveals expression profiles of DEG in FoxO3a-activated and wild-type murine hepatic tissues. Blue to red continuum reflects increased expression abundance. (C) Top 10 significantly altered pathways in FoxO3a-activated hepatic tissues. Bubble size indicates the number of enriched genes; redness denotes significance. qvalue < 0.05 is considered to be statistically significant. (D) GSEA plot showing the enrichment of OXPHOS pathway. NES, normalized enrichment score.

Figure S6

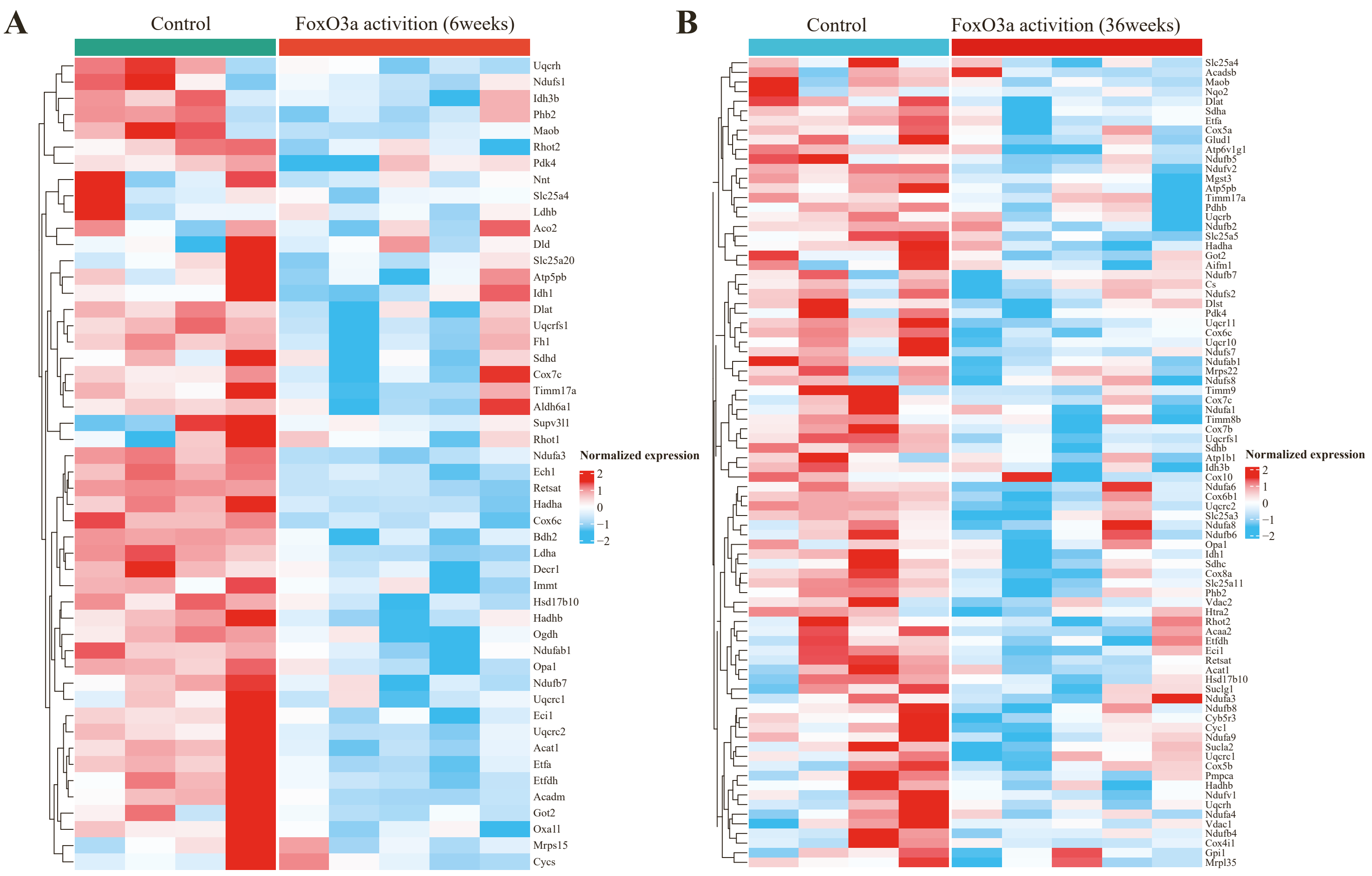

Figure S6. Suppression of OXPHOS pathway genes in FoxO3a-activated hepatic tissues. (A-B) Heatmap visualization of significantly enriched OXPHOS pathway genes in PiZ mice with Foxo3a activation at 6 weeks (A) and 36 weeks (B) of age.

Figure S7

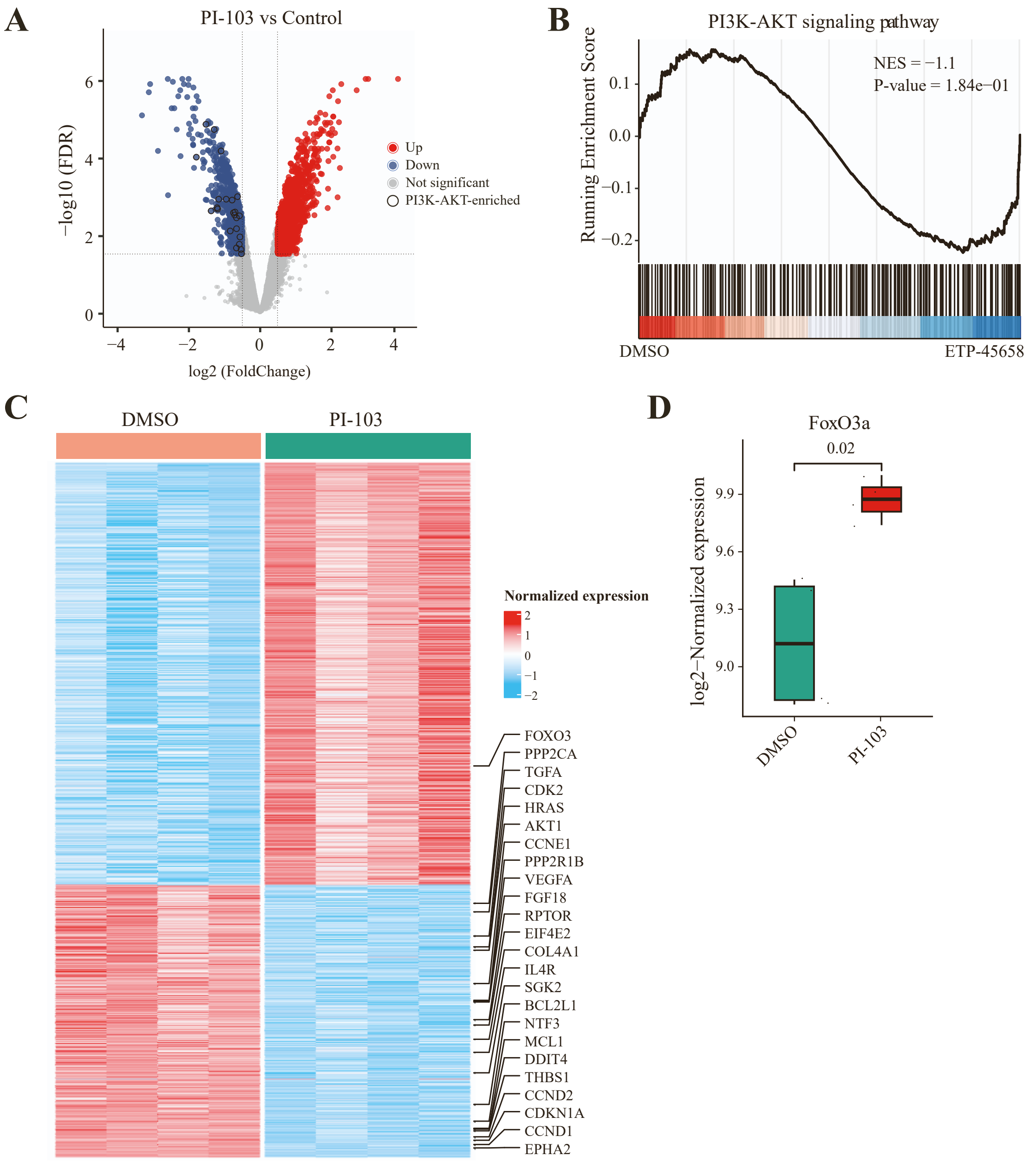

Figure S7. Activation of FoxO3a occurs through the inhibition of the PI3K/AKT pathway mediated by PI-103. (A) Volcano plot illustrating transcriptional changes after PI3K inhibition (PI-103). Differentially expressed genes (DEGs) are identified (FDR < 0.05, |log2FC| > 0.5). Black circles denote PI3K-AKT pathway-enriched genes. Red and blue denote up- and down-regulated genes after PI3K inhibition. (B) Gene set enrichment analysis (GSEA) plot confirming significant suppression of PI3K/AKT signaling following PI-103 treatment. (C) Heatmap shows DEG expression patterns with annotated FoxO3a and core PI3K/AKT genes. Blue to red gradient reflects increased expression abundance. (D) Comparative analysis of FoxO3a expression between PI-103 and DMSO treatment. Box plot elements: center line = median, box limits = first and third quartiles, whiskers = 1.5×interquartile range (IQR).

Supplementary Table 1 Primers used for this study

Primer name	Primer sequences (5'-3')
β-actin-F	TATTGGCAACGAGCGGTTC
β-actin-R	ATGCCACAGGATTCCATACCC
FoxO1-F	CCCAGGCCGGAGTTTAACC
FoxO1-R	GTTGCTCATAAAGTCGGTGCT
FoxO3a-F	CTGGGGGAACCTGTCCTATG
FoxO3a-R	TCATTCTGAACGCGCATGAAG
FoxO4-F	CTTCCTCGACCAGACCTCG
FoxO4-R	ACAGGATCGGTTCGGAGTGT
FoxO6-F	GTGGGGGAACCTTTCCTACG
FoxO6-R	TTCTGCACGCGGATGAACC
Bim-F	GACAGAACCGCAAGGTAATCC
Bim-R	ACTTGTCACAACTCATGGGTG
Bax-F	TGAAGACAGGGGCCTTTTTG
Bax-R	AATTCGCCGGAGACACTCG
Insulin recptor-F	ATGGGCTTCGGGAGAGGAT
Insulin recptor-R	GGATGTCCATACCAGGGCAC
Igf2r-F	GGGAAGCTGTTGACTCCAAAA
Igf2r-R	GCAGCCCATAGTGGTGTTGAA
Igf1r-F	GTGGGGGCTCGTGTTTCTC
Igf1r-R	GATCACCGTGCAGTTTTCCA

cyclin D1-F	CAGAAGTGCGAAGAGGAGGTC
cyclin D1-R	TCATCTTAGAGGCCACGAACAT
cyclin D2-F	GCGTGCAGAAGGACATCCA
cyclin D2-R	CACTTTTGTTCCTCACAGACCTCTAG
Slc25a4-F	AGTTTGACCCTCTCGATCG
Slc25a4-R	AGCTTTCTTAAGGACTTCCTGGCAG
Cox5a-F	TGTCCTTGCTGTGACCTGGGC
Cox5a-R	GAGTCTCCTACACGACTCCAGA
Sdhc-F	AGAAGAGGGGGTGCTAAGATCC
Sdhc-R	GGGCTAGGAGTCCACGCCC
Atp6v0c-F	TTGGACAATTTATGGGCCCTGG
Atp6v0c-R	GGGGACCGATGATCCCAAAAC

Supplementary Table 2. FoxO3a regulon gene list

FoxO3a regulon	Mdga2, Capn12, Izumo1, Myo15, Elp6, Rgs9, Foxo3, Gm19710, Cyp2b10, Gm8579, Ppp2r3a, Btbd16, Plp1, Spag16, Mab21l3, Zfyve1, Dynlt1b, Nos1, Cacnb4, Tbx19, Wipf2, Gpc1, Klc2, Reln, Alk, Cstf2t, Pitpnm2, Rnf123, Tmem181a, Zfp512, Ptpn5 Sema4g, Clec1a, Flcn
